# A synthetic biology approach for evaluating the functional contribution of designer cellulosome components to deconstruction of cellulosic substrates

**DOI:** 10.1186/1754-6834-6-182

**Published:** 2013-12-16

**Authors:** Yael Vazana, Yoav Barak, Tamar Unger, Yoav Peleg, Melina Shamshoum, Tuval Ben-Yehezkel, Yair Mazor, Ehud Shapiro, Raphael Lamed, Edward A Bayer

**Affiliations:** 1 Department of Biological Chemistry, The Weizmann Institute of Science, Rehovot 76100, Israel; 2 Chemical Research Support, The Weizmann Institute of Science, Rehovot 76100, Israel; 3 Structural Proteomics, The Weizmann Institute of Science, Rehovot 76100, Israel; 4 Department of Computer Science and Applied Mathematics, The Weizmann Institute of Science, Rehovot 76100, Israel; 5 Department of Molecular Microbiology and Biotechnology, George S. Wise Faculty of Life Sciences, Tel Aviv University, Ramat Aviv 69978, Israel

**Keywords:** Cellulosomes, Cellulases, Multi-enzyme complex, Cellulosic biomass, Biofuels, *Clostridium thermocellum*

## Abstract

**Background:**

Select cellulolytic bacteria produce multi-enzymatic cellulosome complexes that bind to the plant cell wall and catalyze its efficient degradation. The multi-modular interconnecting cellulosomal subunits comprise dockerin-containing enzymes that bind cohesively to cohesin-containing scaffoldins. The organization of the modules into functional polypeptides is achieved by intermodular linkers of different lengths and composition, which provide flexibility to the complex and determine its overall architecture.

**Results:**

Using a synthetic biology approach, we systematically investigated the spatial organization of the scaffoldin subunit and its effect on cellulose hydrolysis by designing a combinatorial library of recombinant trivalent designer scaffoldins, which contain a carbohydrate-binding module (CBM) and 3 divergent cohesin modules. The positions of the individual modules were shuffled into 24 different arrangements of chimaeric scaffoldins. This basic set was further extended into three sub-sets for each arrangement with intermodular linkers ranging from zero (no linkers), 5 (short linkers) and native linkers of 27–35 amino acids (long linkers). Of the 72 possible scaffoldins, 56 were successfully cloned and 45 of them expressed, representing 14 full sets of chimaeric scaffoldins. The resultant 42-component scaffoldin library was used to assemble designer cellulosomes, comprising three model *C. thermocellum* cellulases. Activities were examined using Avicel as a pure microcrystalline cellulose substrate and pretreated cellulose-enriched wheat straw as a model substrate derived from a native source. All scaffoldin combinations yielded active trivalent designer cellulosome assemblies on both substrates that exceeded the levels of the free enzyme systems. A preferred modular arrangement for the trivalent designer scaffoldin was not observed for the three enzymes used in this study, indicating that they could be integrated at any position in the designer cellulosome without significant effect on cellulose-degrading activity. Designer cellulosomes assembled with the long-linker scaffoldins achieved higher levels of activity, compared to those assembled with short-and no-linker scaffoldins.

**Conclusions:**

The results demonstrate the robustness of the cellulosome system. Long intermodular scaffoldin linkers are preferable, thus leading to enhanced degradation of cellulosic substrates, presumably due to the increased flexibility and spatial positioning of the attached enzymes in the complex. These findings provide a general basis for improved designer cellulosome systems as a platform for bioethanol production.

## Background

Cellulose is the major component of the plant cell wall and is the most abundant renewable source of carbon and energy on Earth. One of the most potent cellulose-degrading microorganisms is the well-studied bacterium, *Clostridium thermocellum*. This anaerobic thermophilic cellulolytic bacterium secretes a multi-enzymatic complex called the cellulosome, first discovered in 1983 [1,2]. Since then, other cellulosomal organisms have been discovered and characterized, which possess an array of cellulosomal architectures [3-16].

Cellulosome architecture is primarily dictated by the non-catalytic scaffoldin subunit. In *C. thermocellum* the scaffoldin is composed of nine repeating cohesin modules, each of which can bind cohesively to a complementary dockerin-module, borne by a cellulosomal enzyme. The scaffoldin targets the cellulosome complex to the cellulose substrate by virtue of an integral carbohydrate-binding module (CBM) [17-19] and attaches to the bacterial cell wall via an alternative type of cohesin-dockerin interaction [20,21]. Enhanced synergism among the catalytic units and close association between the cell-bound cellulosome and substrate serve to minimize diffusion of the enzymes and their hydrolytic products, thus providing the bacterium with a competitive advantage over other organisms [22].

Cellulosome systems in bacteria show great diversity and can be divided into simple and complex systems. Some cellulolytic bacteria, for example, *C. thermocellum, Bacteroides cellulosolvens* and *Acetivibrio cellulolyticus*, have a complex cellulosome system. They produce several types of scaffoldins organized in a scaffoldin gene cluster, in which the genes encoding for the major scaffoldins are clustered together on the chromosome and the genes encoding the cellulosomal enzymes are scattered across the chromosome [23,24]. Another resemblance among these bacteria is that the intermodular linker segments in their primary scaffoldin are relatively long, reaching 20 to 40 residues in length and more, and are rich in proline and threonine residues [25]. In contrast, other clostridial cellulosome-producing bacteria, such as *C. cellulovorans, C. cellulolyticum, C. josui,* and *C. acetobutylicum,* have a simple cellulosome system [4,26-28]. The simple systems contain a single scaffoldin and genes for cellulosomal enzymes are encoded downstream of the scaffoldin gene. The scaffoldins of the simple systems possess markedly shorter linkers than those of the complex systems. For example in *C. cellulovorans* the linkers of scaffoldin CbpA range between five and eight residues. Interestingly, in some scaffoldins - even those of complex systems - neighboring cohesins may not be separated by linkers at all, such as the first and second or the third and fourth cohesins in ScaB from *B. cellulosolvens*. At the other extreme are linkers as long as 100 to 700 residues [25]. The lengths and composition of the intermodular linker segments are not arbitrary, and their content and disposition likely play a relevant role in cellulosome function.

The position of the CBM in the scaffoldins also differs between the cellulosome systems. In the simple systems the CBM is invariably positioned at the N-terminus of the scaffoldin and is followed by one or more *X*2 modules [29]. In contrast, the complex systems are characterized by an internal CBM, surrounded on both the N- and C-terminal sides by cohesins.

Designer cellulosomes are composed of an artificial chimaeric scaffoldin, and a set of cellulases [20,30]. The synthetic scaffoldin consists of a CBM module, which targets the entire complex to the cellulosic substrate, together with several cohesin modules, derived from different species, that have divergent specificities. In turn, the cellulases each have a complementary and specific dockerin-module, which binds selectively to one of the divergent cohesins [31-33]. Unlike native cellulosome systems, the use of designer cellulosomes thus enables control over the composition and the positions of the chosen cellulases within a given mini-cellulosome, thereby allowing the production of homogeneous complexes. In this context, the controlled incorporation of cellulases into artificial designer cellulosomes was shown to induce enhanced synergism between cellulases via their targeting to the substrate by the family-3a scaffoldin-borne CBM module, or by the proximity of the cellulases in the complex [34-43]. In this study, we employed a synthetic biology (SynBio) approach by preparing an extensive combinatorial library of appropriate trivalent chimaeric scaffodins in order to address the question of whether enzyme position within the cellulosome complex and/or length of intermodular linker contribute/s to cellulosome performance. The latter scaffoldins enabled precise integration of three different cellulases in distinct spatial and positional arrangements relative to an integral CBM. The resultant designer cellulosomes were examined for activity on recalcitrant microcrystalline cellulose and pretreated cellulose-enriched wheat-straw substrates.

## Results

### Design and construction of designer-cellulosome subunits

#### Chimaeric scaffoldins

**Figure 1:**
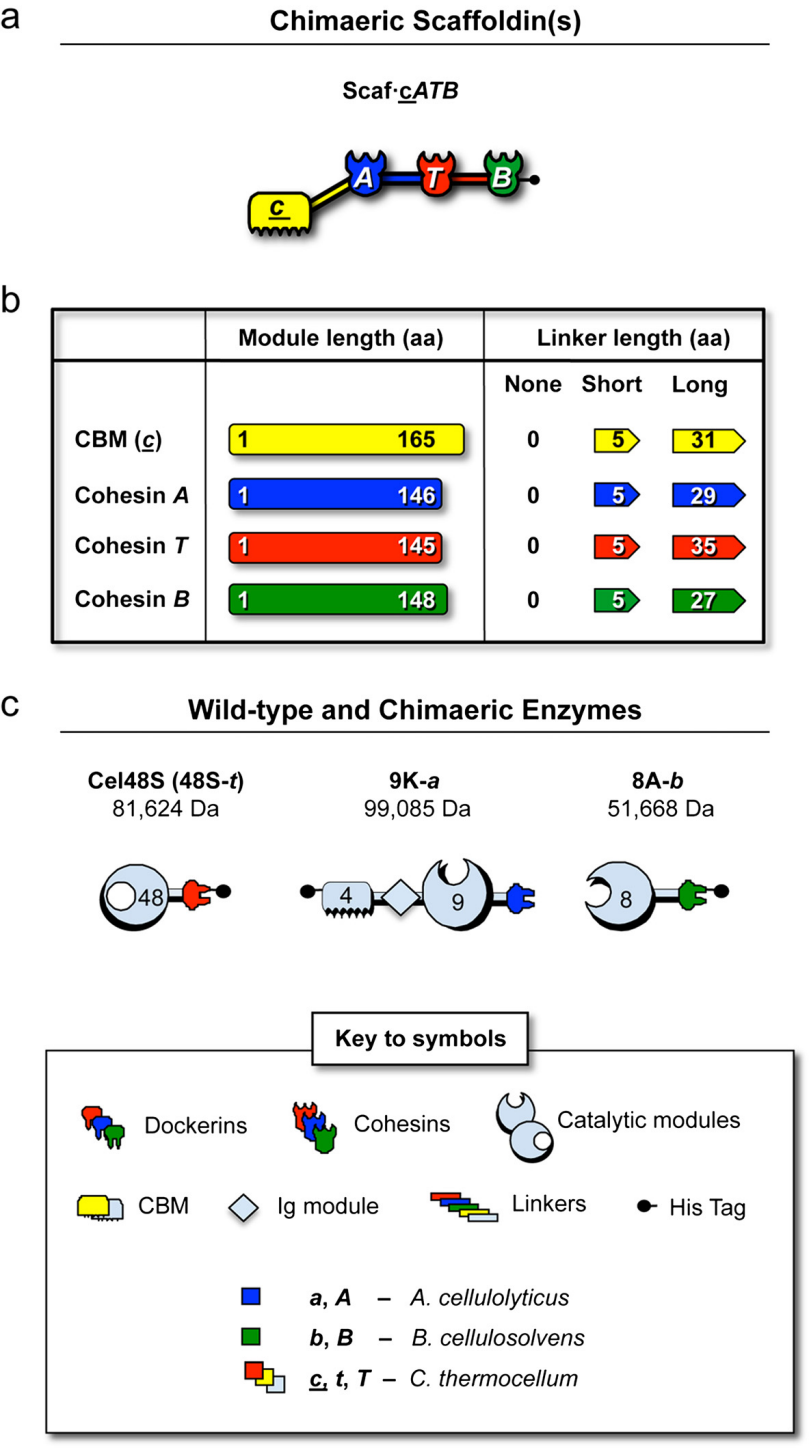
**Schematic representation of the recombinant proteins used in this study.** The modular notation, structure and molecular mass of each protein are indicated. Red, yellow and light blue indicate *C. thermocellum*-derived cohesin/dockerin, carbohydrate binding module (CBM) and enzyme-related components, respectively. Dark blue indicates *A. cellulolyticus*-derived cohesin/dockerin modules, and green indicates *B. cellulosolvens*-derived modules. (**a**) The basic chimaeric scaffoldin containing three divergent cohesins: the third cohesin of scaffoldin ScaC from *A. cellulolyticus* (***A***), the third cohesin of ScaB from *B. cellulosolvens* (***B***), the second cohesin of the CipA scaffoldin from *C. thermocellum* (***T***) plus a CBM3a module of the same scaffoldin (***c***). See Additional file 1: Table S1 for the molecular weights of the respective chimaeric scaffoldins. (**b**) The length of each module and its C-flanking linkers in amino acid residues. (**c**) Recombinant cellulases used in this study. In the modular notation of the enzymes, the number indicates the GH family of the catalytic domain. S, K and A indicate the original name of the enzyme (Cel48S, Cel9K and Cel8A, respectively). The chimaeric Cel9K includes a CBM4 and Ig domain on the N-terminal portion of the enzyme. Lowercase *t, a* and *b* indicate the source of the dockerin module (*C. thermocellum*, *A. cellulolyticus* and *B. cellulosolvens* respectively).

In this work, we designed a basic scaffoldin template with three divergent cohesin modules of different specificities and a CBM module, in which all of the modular components are separated by linker segments (Figure 1; Additional file 1: Table S1). The origins of the modules that were used to create the scaffoldin library included the second cohesin and the CBM3a of CipA from *C. thermocellum*, (designated *T* and *c* respectively, the third cohesin of ScaB from *B. cellulosolvens* (designated *B*) and the third cohesin of ScaC from *A. cellulolyticus* (designated *A*). All three of these bacteria produce a complex cellulosome system, bearing several interconnecting scaffoldin subunits.

In order to study the importance of the spatial organization of the cellulases in the scaffoldin we used different intermodular linker lengths between the cohesin and the CBM modules, thereby imitating the diversity that exists in nature - long 27 to 35 amino-acid intermodular linkers (simulating the native linkers of *C. thermocellum, A. cellulolyticus, B. cellulosolvens* and *R. flavefaciens*), short 5 amino-acid linkers (emulating the linkers of *C. cellulovorans, C. cellulolyticum, C. josui,* and *C. acetobutylicum*) or without such linkers (designated no or none) (Figure 1b).

In order to produce long-linker scaffoldins, each of the modules (CBMs and cohesins) was cloned together with its C-terminal native linker (approximately 30 residues), except for the (third) *B. cellulosolvens* cohesin, which lacks such a linker. Since we have used this particular *B. cellulosolvens* cohesin-dockerin pair routinely and successfully in our lab for designer-cellulosome studies, we thus attached another *B. cellulosolvens* linker (C-terminal to the fourth cohesin in the native scaffoldin) in its stead. For the short linkers, we used only the first five residues of each of the native linkers, and for scaffoldins that lack linkers, the modules were simply combined without them. The amino-acid content of the different linkers used in this work is shown in Table 1.

**Table 1:** The set of inter-modular linkers used for cloning

**Source organism**	**Scaffoldin**	**Module**	**C-terminal linker**	**Length (a.a)**	**Sequence**	**Accession code**
*A. cellulolyticus*	ScaC	Coh ***A***	Long	29	PTPTQSATPTVTPSATATPTQSATPTVTP	AAP48996
			Short	5	PTPTQ	
			None	—	—	
*B. cellulosolvens*	ScaB	Coh ***B***	Long	27	TPTNTISVTPTNNSTPTNNSTPKPNPL	AAT79550
			Short	5	TPTNT	
			None	—	—	
*C. thermocellum*	CipA	Coh ***T***	Long	35	PTKGATPTNTATPTKSATATPTRPSVPTNTPTNTP	ABN54273
			Short	5	PTKGA	
			None	—	—	
		CBM (***c***)	Long	31	VVPSTQPVTTPPATTKPPATTKPPATTIPPS	ABN54273
			Short	5	VVPST	
			None	—	—	

The preceding module of each linker is indicated. Coh ***A:*** the third cohesin of ScaC from *A. cellulolyticus*, Coh ***B*:** the third cohesin of ScaB from *B. cellulosolvens,* Coh ***T:*** The second cohesin of CipA from *C. thermocellum*, CBM (***c***): the carbohydrate binding module CBM3a of CipA from *C. thermocellum*.

The four modules (three divergent cohesin modules and a CBM) could thus be shuffled, resulting in twenty-four different arrangements, each with linkers of three different lengths separating the modules. Therefore, from the basic scaffoldin template, 72 possible combinations could potentially be produced. The cloning of this scaffoldin set, however, proved to be a challenging task, mainly due to the repetitive composition of the intermodular linkers, the length of the DNA encoding the recombinant scaffoldins (approximately 2,100 bp), and the relatively large number of constructs.

Two different approaches were used for cloning: in the first approach, a computer-aided, automated method for combinatorial DNA library design and production was employed for construction and cloning of the scaffoldins that either lacked intermodular linkers or contained short intermodular linkers. The design and synthesis of scaffoldins using this approach were performed using computer-aided methods for specifying, visualizing, planning and executing the actual production of the desired DNA libraries [44,45]. This approach, however, ultimately proved inadequate for cloning the scaffoldins containing the long intermodular linkers, presumably due to the highly repetitive nature of the DNA sequence of these constructs.

**Figure 2:**
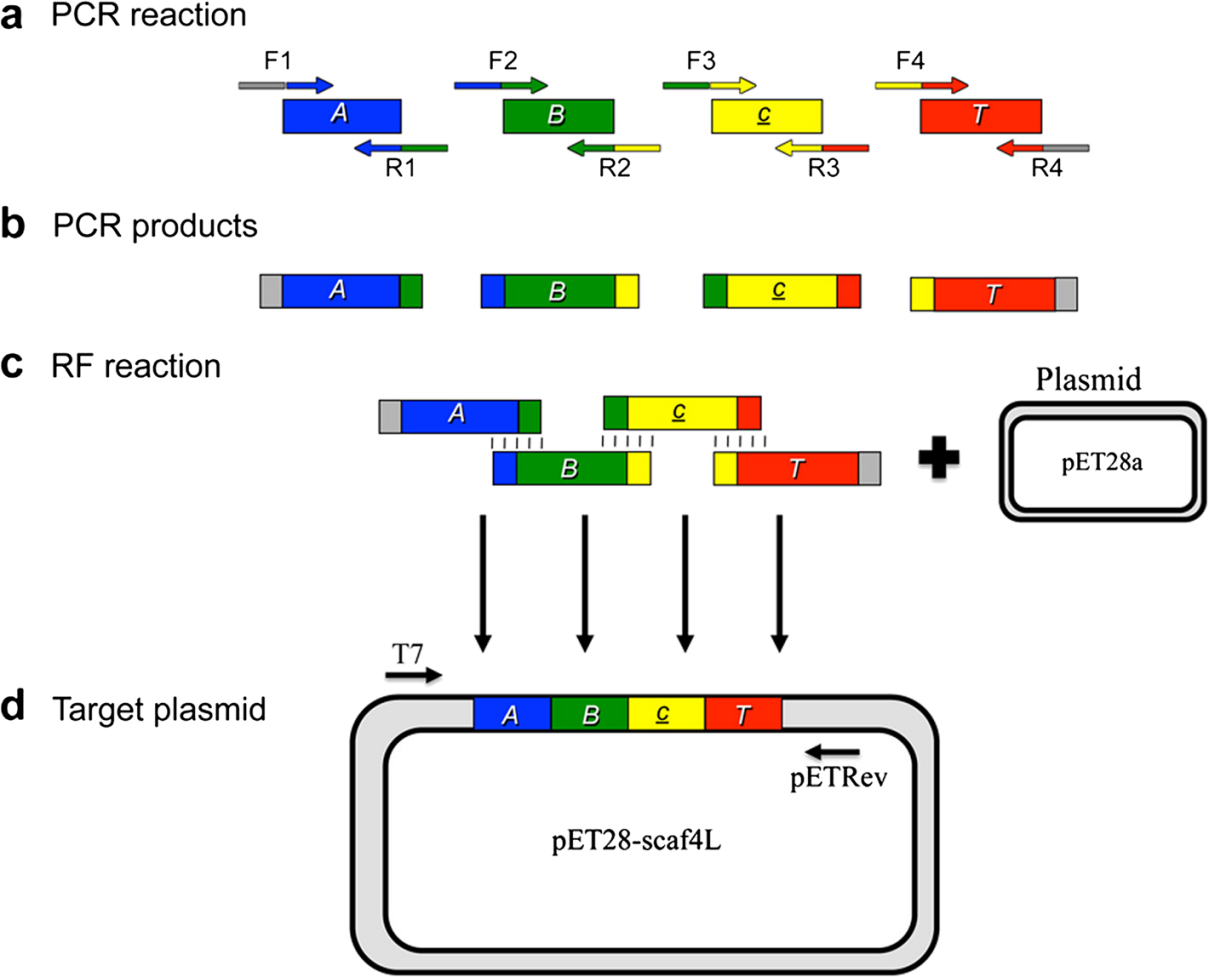
**Multi-component assembly of a chimaeric scaffoldin - ‘Scaf4L’.** (**a**) Schematic representation of the four components that were amplified in the first PCR reaction: *A. cellulolyticus* cohesin (***A***) and C-terminal linker segment, *B. cellulosolvens* cohesin (***B***) and linker, carbohydrate binding module (CBM) (***c***) and linker and *C. thermocellum* cohesin (***T***) but without the C-terminal linker. (**b**) Resultant PCR products. (**c**) The PCR products were then used as mega-primers for the restriction-free reaction with the pET28a plasmid. The sequences that form base-pairing due to flanking regions designed in the primers of each module are indicated by the coloring of the PCR products and by connecting lines. (**d**) The resulting linear plasmid pET28a-scaf4L. Ligation occurs spontaneously in *E. coli*.

**Figure 3:**
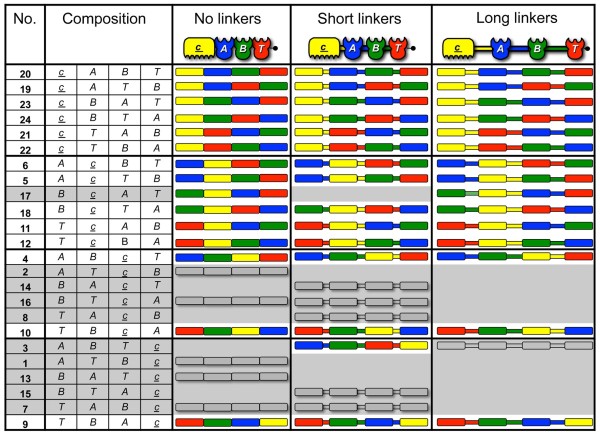
**Schematic representation of the scaffoldins in the final scaffoldin library.** Twenty-four different arrangements of the cohesin (***A, B*** and ***T***) and carbohydrate binding module (CBM) (***c***) modules are shown in three sub-libraries: no-linker, short-linker and long-linker versions of the given chimaeric scaffoldins. The left column indicates the number of each scaffoldin set according to its composition (position of CBM and divergent cohesins). The 45 successfully cloned and expressed scaffoldins included in the final library are shown as colored pictograms. An additional 11 scaffoldins were cloned but not expressed (shown as gray pictograms); 16 additional scaffoldins were not expressed (gray background); 14 full sets, representing 42 cloned and expressed scaffoldins, were finally achieved for further study.

We therefore applied a second approach for cloning the scaffoldins with long intermodular linkers that involved restriction-free multi-component assembly of the DNA segments. Plasmids for 16 long-linker trivalent scaffoldins were thus constructed by employing simultaneous restriction-free cloning [46]. For construction of each scaffoldin, eight primer pairs were designed. A His-tag was added at the C terminus of each construct to promote subsequent purification using a Ni-nitrilotriacetic acid (NTA) column (Qiagen GmbH, Hiden, Germany). The four modules were amplified by PCR with 25- to 30-bp overhangs on both the 5′ and 3′ ends, corresponding to the adjoining regions (either with another adjoining insert gene or with the expression vector as needed). Next, the PCR products served as mega-primers for simultaneous assembly of the vector and inserts by linear amplification. The assembly of a representative scaffoldin with the following modular arrangement: Scaf · *ABcT*, bearing long linkers is depicted in Figure 2. This particular scaffoldin gene is herein designated *scaf4L*, where the number indicates the arrangement of modules as it appears in Figure 3, and the letter L indicates the length of the intermodular linkers (L indicates long linker, S short, and N no linkers).

In the end, 56 of the 72 scaffoldins were successfully cloned using the two approaches, and of these, 45 were expressed successfully in an *E. coli* host-cell system (Figure 3). Special efforts were spent in attempts to complete full sets, although certain sets were eventually abandoned after numerous trials. Specifically, sets 3 and 17 remained unfinished, owing to difficulties in cloning or expression of the residual constructs. The final scaffoldin library used for further study included 14 full sets, consisting of 42 different trivalent chimaeric scaffoldins (Figure 3). In seven of these sets the CBM was located in an internal position: in sets 5, 6, 11, 12, and 18 the CBM occupied the second position, and in sets 4 and 10 the CBM was in the third position. In the remaining seven completed sets, the CBM was located at the extremities: that is, the C-terminal position in set 9 and the N-terminal position in sets 19 to 24. The latter sets with the CBM at the N terminus represented a complete collection of six scaffoldins with the different cohesins located in all possible combinations.

#### Matching dockerin-bearing cellulases

**Figure 4:**
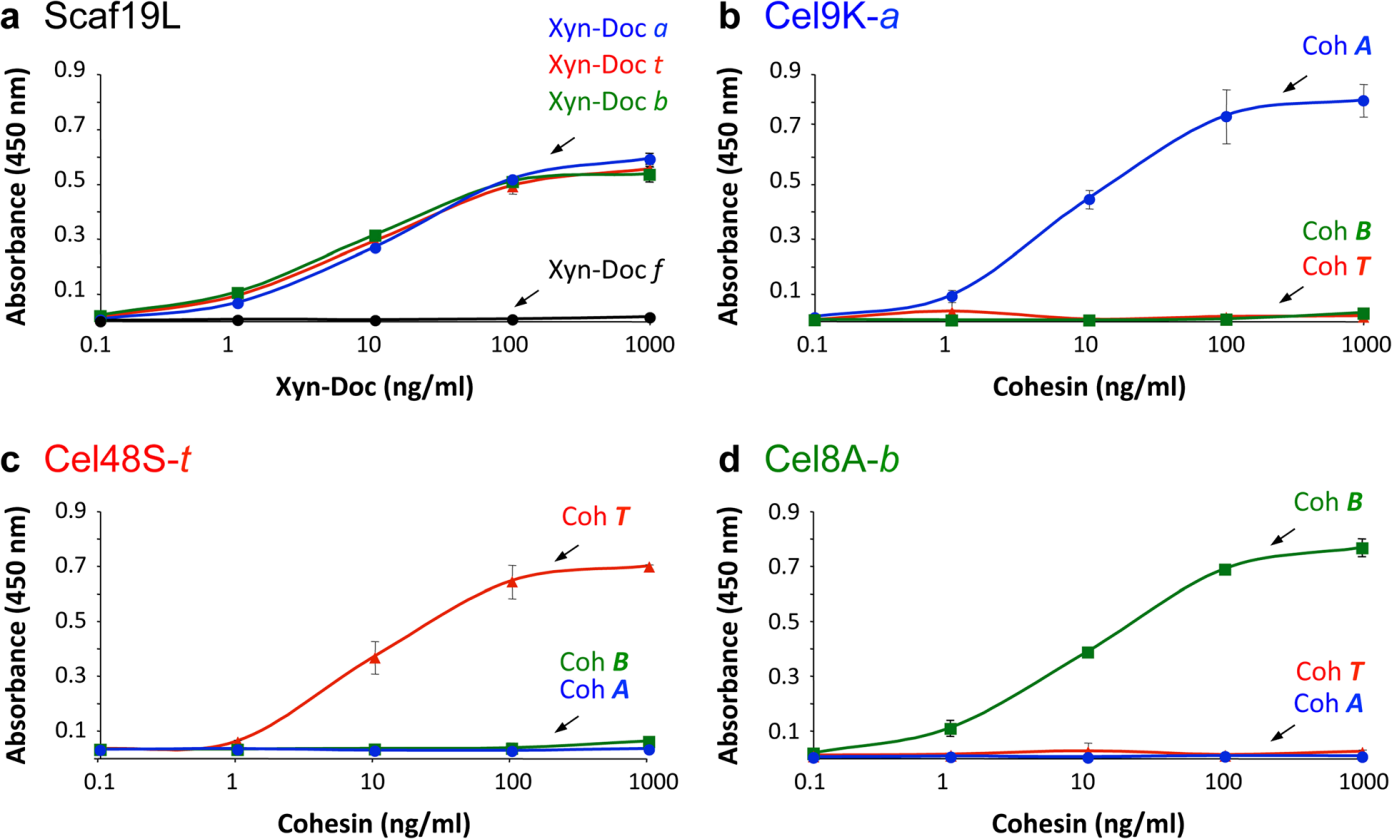
**Specificity of the cohesin-borne scaffoldin for its matching dockerins (a) and of the dockerin-bearing enzymes for their target cohesins (b-d).** The interaction between scaffoldin 19L (Scaf19L) and its matching dockerins was examined (**a**) using a standardized matching fusion-protein system [73]. Scaf19L was coated onto the wells of a microtiter plate and was subjected to interaction with Xyn-Doc fusion proteins, Xyn-Doc *t* (red), Xyn-Doc *a* (blue), Xyn-Doc *b* (green), and a non-matching control Xyn-Doc *f,* the divergent dockerin of which was derived from a cellulosomal component of the rumen bacterium, *Ruminococcus flavefaciens* (black). Anti-xylanase antibodies were used, together with a second antibody-conjugated enzyme system to promote a chromogenic reaction. Similarly, the interaction between the enzymes via their dockerin module to matching and non-matching cohesins was examined. In this case, 9K-*a* (**b**), 48S-*t* (**c**) and 8A-*b* (**d**), which bear an *A. cellulolyticus*, *C. thermocellum* and *B. cellulosolvens* dockerin, respectively, were coated onto the wells of microtiter plates and subjected to interaction with the designated carbohydrate binding module (CBM)-fused cohesin modules, Coh ***T*** (red), Coh ***A*** (blue) and Coh ***B*** (green).

Three prominent cellulases from *C. thermocellum* were used in combination with the chimaeric scaffoldins: an exoglucanase Cel48S, an endoglucanase Cel8A and the multimodular endoglucanase Cel9K [47-55]. The Cel48S exoglucanase was used together with its native dockerin. For subsequent self-assembly of designer cellulosomes using the library of chimaeric scaffoldins, the catalytic module of *C. thermocellum* endoglucanase Cel8A was fused to the divergent *B. cellulosolvens* ScaA dockerin and the three Cel9K modules were fused to the divergent *A. cellulolyticus* ScaB dockerin. For the purposes of the present work, the resultant cellulases were designated 48S-*t*, 8A-*b* and 9K-*a,* respectively, where 48S, 8A and 9K represent the catalytic module of the corresponding enzyme (9K also includes the CBM4 and Ig module) and *t, b* and *a* represent the bacterial source of the dockerin module (see Figure 1c). The specificity of the cohesin-dockerin interaction was verified by affinity-based ELISA (Figure 4). Each enzyme was shown to interact exclusively with its matching cohesin and not with the two other divergent cohesins that are present in the designer scaffoldin set (Figure 4b-d). The resultant recombinant enzymes were then tested for their ability to degrade phosphoric acid-swollen cellulose (PASC) or Avicel, and their activities were comparable to those of the wild-type enzymes. In addition, the enzymes were interacted with beaded cellulose and found to bind cellulose either by their dockerin-fused catalytic module alone (8A-*b*, 48S-*t*) and/or by an inherent CBM4 module in the case of 9K-*a,* known to bind non-crystalline (PASC) and microcrystalline forms of cellulose [56] (Additional file 2: Figure S1).

#### Purification schemes

A general method for the expression and purification of the 42 scaffoldins was designed based on a scheme devised initially for one of the chimaeric scaffoldins (Scaf9S). Interestingly, a heat-treatment step during the purification protocol, which precipitated the bulk of the native *E. coli* proteins, did not damage the scaffoldin even though two of the cohesin modules, which account for half of the cohesins in the chimaeric scaffoldin (that is, *A. cellulolyticus* and *B. cellulosolvens*) were of mesophilic origin. The scaffoldin was further subjected to a two-stage affinity purification procedure, based on its binding to cellulose via the CBM module and to an Ni-NTA column by virtue of its His-tag. This two-step strategy allowed us to purify mostly intact scaffoldins, since almost all of the scaffoldins (apart from scaffoldin 9, 10 and 4) had the CBM module at the N-terminus or relatively close to the N-terminus of the protein, whereas the His-tag was appended to the scaffoldin on the opposite C-terminus part. In this manner we avoided purifying scaffoldins that were proteolytically cleaved during expression, since the truncated forms lost one of the tags that was used for purification. Subsequently, the remaining 41 scaffoldins were expressed and purified accordingly in batch, 2 to 4 scaffoldins at a time.

The purity of the scaffoldins was analyzed using SDS-PAGE. In initial purification experiments, two of the scaffoldins seemed to be truncated, since they migrated as two separate bands on the gel. Scaffoldin ‘9L’ with the following modular composition: Scaf · *TBAc* showed 72- and 55-kDa bands (data not shown). The 72-kDa band corresponded to that of the intact scaffoldin, and the 55-kDa band was consistent with a scaffoldin without the first *C. thermocellum* cohesin. Indeed, non-denaturing PAGE of the complex of this scaffoldin showed a shifted band corresponding to the designer-cellulosome complex and a lower band of excess 48S-*t*, which could not bind to the *C. thermocellum* that lacked the matching cohesin. The second truncated scaffoldin, Scaf10L (Scaf · *TBcA*), showed a similar pattern. In order to overcome this problem, we transferred the His-tag of these two scaffoldins from the C to the N terminus. In this manner we could purify the intact scaffoldins, because in the first step the full-length scaffoldin is bound to cellulose via its C-terminal CBM and in the second step via its N-terminal His-tag to the nickel column. Using this approach, all of the purified scaffoldins showed a principal band, which corresponded to the full-length uncleaved protein (Additional file 3: Figure S2). The specificity of the cohesin-dockerin interactions was then verified by affinity-based ELISA. For example, scaffoldin19L was shown to interact with its three matching dockerins *C. thermocellum*, *B. cellulosolvens* and *A. cellulolyticus* but not with the non-matching *R. flavefaciens* dockerin (Figure 4A).

### Analysis of designer-cellulosome assembly

**Figure 5:**
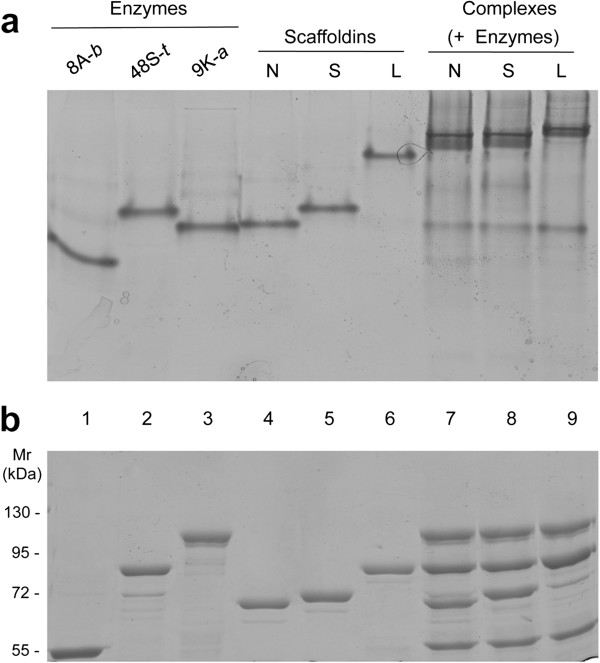
**Electrophoretic mobility of components and assembled complexes on non-denaturing PAGE (a) and SDS-PAGE (b).** Equimolar concentrations of the enzymes 48S-*t*, 9K-*a* and 8A-*b* (lanes 1 to 3 respectively) and the matching scaffoldins without intermodular linkers, with short and long intermodular linkers (Scaf19N, Scaf19S and Scaf19L designated as: N, S and L, lanes 4 to 6, respectively) were interacted to form the respective designer cellulosome complexes (lanes 6 to 9). Mr, molecular mass marker.

The formation of designer-cellulosome complexes was initially analyzed in each case by non-denaturing PAGE. Molar ratios for complete interaction of each enzyme were first determined with several representative scaffoldins from the scaffoldin set (data not shown). These predetermined molar ratios were used for the interaction of the three enzymes with the entire 42-scaffoldin set, and non-denaturing PAGE was used to evaluate the resultant complexes. Each complex migrated on the gel as a major band, shifted from the bands of the individual components of the designer cellulosome, indicating a productive near-complete or complete interaction in each case. A representative example for chimaeric scaffoldin set 19 (*cATB*) is shown in Figure 5.

**Figure 6:**
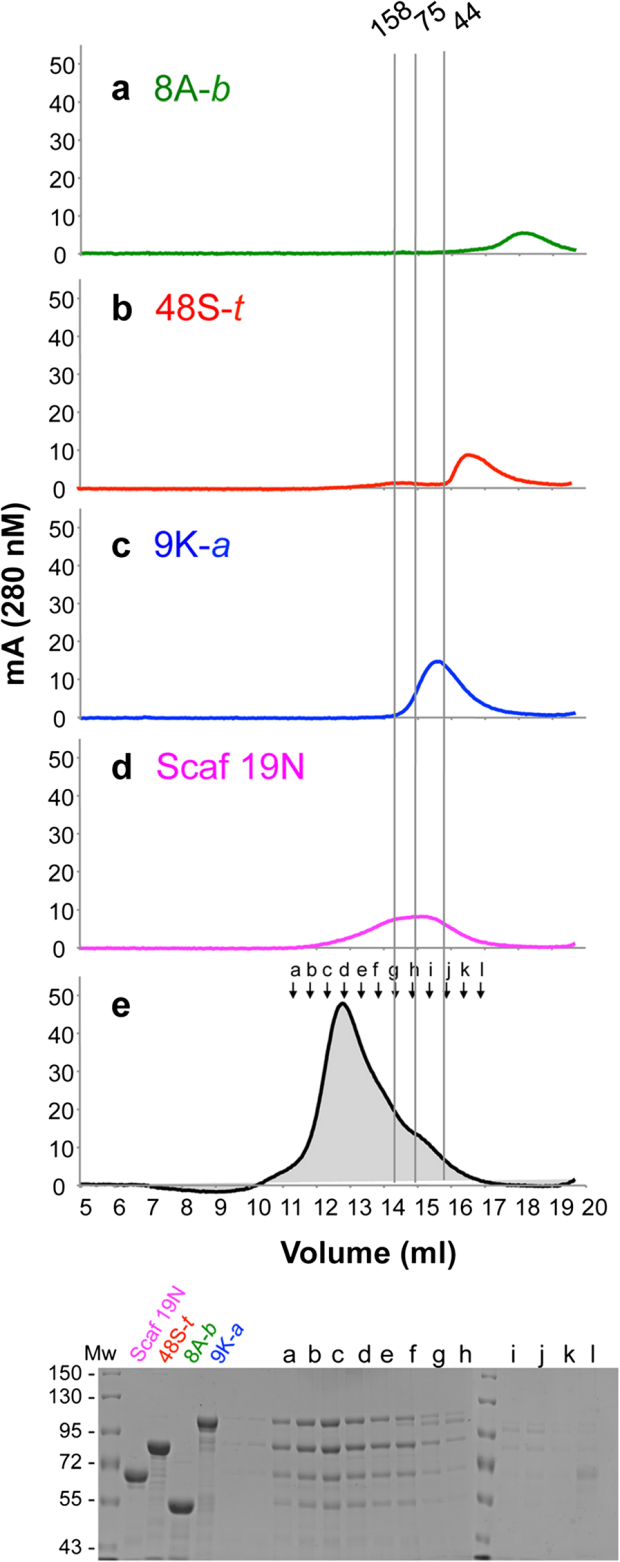
**Superdex 200 gel filtration fast protein liquid chromatography (FPLC) elution profile of a designer-cellulosome complex composed of three chimaeric *C. thermocellum *enzymes, 9K-*a, *48S-*t *and 8A-*b, *assembled on a trivalent chimaeric scaffoldin (Scaf19N) without intermodular linkers.** The elution profile of each of the single components was used as a marker. The curves are labeled as follows: (**a**) 8A-*b*: green, 51.6 kDa, (**b**) 48S-*t*: red, 81.6 kDa|, (**c**) 9K-*a*: blue, 101.4 kDa, (**d**) scaffoldin 19N: magenta, 66 kDa, and (**e**) designer cellulosome complex: black with gray filling. The gel on the bottom shows the SDS-PAGE analysis of the designated elution fractions of the designer cellulosome complexes.

In addition, the designer cellulosome complexes were analyzed by size exclusion chromatography (Figure 6), whereby each of the single components was assessed separately, and their retention volume was used as markers for analysis of the designer cellulosome complexes. The order in which the cellulases eluted from the column corresponds to their molecular masses: 9K-*a* (101.4 kDa), 48S-*t* (81.6 kDa) and 8A-*b* (51.6 kDa). However, their elution profiles were retarded in comparison to their absolute calculated molecular weights. This property has been reported earlier, and the impeded mobility was suggested to stem from the presence of the dockerin module, as the catalytic module by itself elutes as expected [34,57,58]. Scaffoldins 19N, 19S and 19L (66, 67.5 and 75.3 kDa, respectively), however, eluted at a higher retention volume than expected (before 9K-*a*), possibly due either to extended non-globular folding or to dimerization [34,57,58]. The designer cellulosome complexes eluted significantly faster than the single enzymes and scaffoldins as a major peak with a small shoulder that may indicate an excess of an enzyme or a scaffoldin, or, alternatively, a population of designer cellulosomes in which an enzyme is missing. The elution of the 19L designer cellulosome assembled with the long-linker scaffoldin was faster than that of the designer cellulosome assembled with the no-linker and short-linker scaffoldins (19N and 19S), which eluted at similar volumes (not shown). Fractions from the designer cellulosome complexes were pooled and concentrated and then analyzed by SDS-PAGE (Figure 6, bottom). The major peak was shown to consist of all three enzymes together with the chimaeric scaffoldin.

### Activity of designer cellulosomes with selected *C. thermocellum* cellulases

#### Kinetics, synergy, proximity and targeting of the C. thermocellum enzymes Cel48S, Cel8A and Cel9K

The three enzymes used in this study, Cel48S, Cel9K and Cel8A are major components in the *C. thermocellum* cellulosome and are up-regulated when *C. thermocellum* is grown on crystalline cellulose as a carbon source [47-54]. Consequently, we employed as substrates the commonly used commercially available Avicel and a pretreated, cellulose-enriched wheat straw preparation. The final composition of the pretreated wheat straw was determined to be 90% cellulose, 5% hemicellulose and 5% lignin.

**Figure 7:**
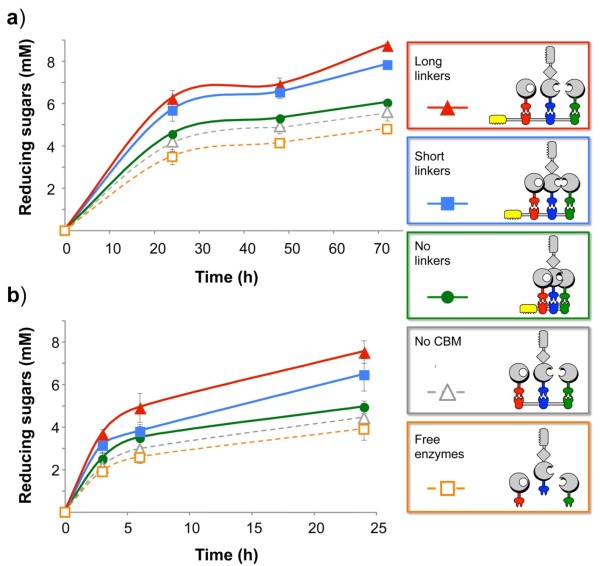
**Kinetics of Avicel (a) and pretreated cellulose-enriched wheat straw (b) hydrolysis by designer-cellulosome complexes and free enzymes.** The graphs show degradation by scaffoldin-set 21 with the following modular organization ***cTAB***: long-linker scaffoldin-based designer-cellulosome (red), the short linker-based designer cellulosomes (blue), and the designer cellulosomes based on the scaffoldin without intermodular linkers (green). Controls include degradation by a designer cellulosome containing a scaffoldin that lacks a carbohydrate binding module (CBM) (gray) and degradation by the free enzymes (orange). Enzymatic activity was defined by release of reducing sugars (mM) as determined by a glucose standard curve. All reactions were carried out in triplicate. Standard deviations from three separate experiments are indicated.

In order to determine the endpoint for the cellulose hydrolysis reaction on either substrate, we initially performed kinetics assays (Figure 7). Enzyme kinetics were tested with three representative scaffoldins of the following modular composition: Scaf · *cTAB* (Scaf21), with the three different linker lengths (none, short and long linkers). In addition, a control scaffoldin lacking a CBM module, with long intermodular linkers and the same modular composition (Scaf · *TAB*), was used as a substrate-targeting control. The free enzymes, not attached to a scaffoldin, were also used as a control. The kinetics of hydrolysis were determined with pretreated wheat straw and Avicel at three timepoints (24, 48 and 72 h for Avicel (Figure 7a) and 3, 6 and 24 h (Figure 7b) for the wheat-straw substrate) until the hydrolysis reaction reached 7.8% degradation of Avicel after 72 h and 40% of the pretreated wheat straw after 24 h. We originally suspected that emplacement of the CBM module may have an effect on the level of activity. Therefore, in addition to designer cellulosomes assembled using Scaf · *cTAB* (Scaf21) with an N-terminal CBM (Figure 7), we also examined two additional scaffoldin sets, Scaf · *TBAc* (Scaf9) with a C-terminal CBM and Scaf · *TcBA* (Scaf12) with an internal CBM (data not shown). The kinetics of the three full sets were very similar with respect to yields and slope. On this basis, we used the same endpoint for the rest of the scaffoldins.

The activity of the individual enzymes was tested on both substrates and was compared to that of the combined enzymatic activity in order to determine the apparent synergy between them (Additional file 4: Table S2). The three enzymes displayed 2-fold synergistic activity on pretreated wheat straw but no synergy was observed on Avicel. The overall enhancement in activity of the free enzymes compared to the enzymes bound to a long-linker designer scaffoldin was 1.82-fold for Avicel and 1.93-fold for pretreated wheat straw. The activities of the designer scaffoldins increased with the increase in linker length on both substrates, by 1.4-fold from the no-linker to the long-linker scaffoldin-mediated complex. The proximity effects were determined by comparing the activity of the combined free enzymes to the enzymes with the control scaffoldin, and were 1.17 and 1.14 for Avicel and pretreated wheat straw, respectively. The targeting effect was determined to be 1.56-fold for Avicel and 1.7-fold for pretreated wheat straw by comparing the activities of the enzymes with a designer scaffoldin with the long intermodular linkers, to the activity of the control scaffoldin lacking the CBM (Figure 7).

#### Effect of spatial organization of the scaffoldin on activity

Next, we tested for cellulose hydrolysis by designer cellulosomes composed of each of the scaffoldins in the library, at a single timepoint (pre-determined by the kinetics assay). For Avicel, activity was tested at 72 h, since shorter incubation times had lower than 5% conversion rates. For pretreated wheat straw the kinetics reaction reached a conversion of about 20% after 3 h; thus longer incubation times were unnecessary. Designer cellulosomes, comprising members of the fourteen different scaffoldin arrangements (sets), were tested on Avicel and pretreated wheat straw for their activities in combination with the three cellulases; for each arrangement we tested three scaffoldins that vary in the length of the intermodular linkers from 0 (no linkers), 5 (short linkers) to an average of 30.5 (27 to 35, long-linker) amino acids, respectively (Figure 1).

**Figure 8:**
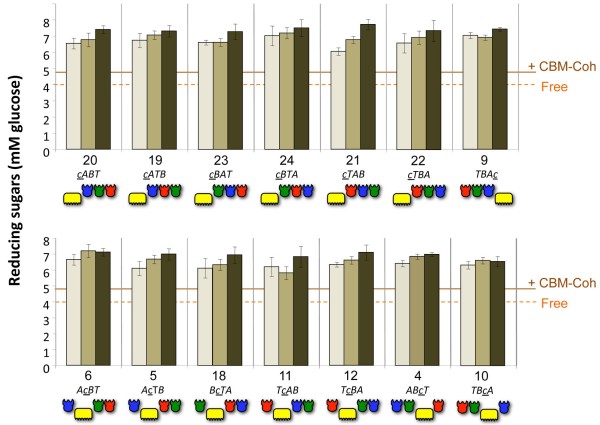
**Comparative hydrolysis of Avicel by the 14 sets of designer cellulosomes.** The modular composition of each set and the scaffoldin number is denoted on the x-axis. Upper panel: the carbohydrate binding module (CBM) of the designer scaffoldin is at one of the terminal positions (N or C terminus). Lower panel: the CBM module of the designer scaffoldin is in an internal position. Each designer-cellulosome set is assembled either without intermodular linker scaffoldin (light brown), with short intermodular linker scaffoldin (medium brown), and with long intermodular linker scaffoldin (dark brown). Controls: Free: corresponds to the combined activity of 48S-*t*, 9K-*a* and 8A-*b.* CBM-Coh represents a cellulose-targeting control, corresponding to the activity of the three dockerin-bearing enzymes, each attached separately to its matching cohesin module fused to a CBM. Reactions were carried out for 72 h. Enzymatic activity was defined by mM reducing sugars as determined by a glucose standard curve. All reactions were carried out in triplicate and repeated three times. Standard deviations of at least three experiments are indicated.

**Figure 9:**
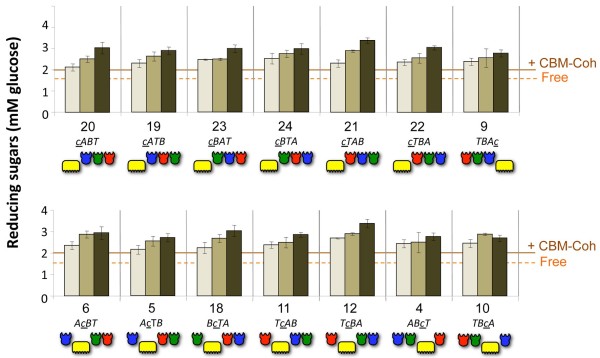
**Comparative hydrolysis of pretreated cellulose-enriched wheat straw by the 14 sets of designer cellulosomes.** Reactions were carried out for 3 h on pretreated cellulose-enriched wheat straw. All other details are provided in the legend to Figure 8.

The results are displayed in Figure 8, using Avicel as a commercially available model microcrystalline cellulose substrate, and in Figure 9, using pretreated wheat straw as a cellulose-enriched substrate from a natural source. In both Figures 8 and 9, the upper panels provide the results of cellulosomes with scaffoldins bearing CBMs at the extremities and the lower panels show the activities of the cellulosomes having scaffoldin sets with internal CBMs.

All of the designer cellulosomes synthesized in this work acted synergistically and showed substantially higher activities on both substrates compared to those of the combined free dockerin-bearing enzyme systems, whether fitted with matching CBM-fused cohesins (CBM-Cohs) or not. When the three enzymes were in the free state, they degraded Avicel (Figure 8) at a level that produced about 4.0 mM reducing sugars (glucose equivalents); when each enzyme was combined with a matching CBM-Coh for substrate targeting, the observed Avicel-degrading activity increased to about 4.8 mM glucose equivalents. In contrast, the observed activities of the designer cellulosomes ranged from approximately 5.8 to approximately 7.7 mM glucose equivalents after a 72-h reaction period. The lowest value was observed for the designer cellulosome based on the short-linker scaffoldin Scaf · *TcAB* (Scaf11S, set 11) with an internal CBM, and the highest value was achieved for the designer cellulosome with the long-linker scaffoldin Scaf · *cTAB* (Scaf21L, set 21). The substantial increase in cellulolytic activity on the microcrystalline cellulose substrate indicates that all of the different combinations of designer cellulosomes exhibited enhanced synergistic activities among the enzymes, irrespective of the position of the enzyme or CBM in the complex.

Equivalent results were observed on the pretreated wheat-straw substrate (Figure 9). The observed activities on this substrate ranged from approximately 2.1 to 3.5 mM reducing sugars following a 3-h reaction period, compared to 1.5 and 2.0 of the free enzymes without and with matching CBM-Cohs, respectively. The lowest value was observed for the no-linker scaffoldin Scaf · *cABT* (Scaf20N, set 20, N-terminal CBM), and the highest value for the long-linker scaffoldin Scaf · *cTAB* (Scaf12L, set 12, internal CBM).

The results (Figures 7, 8 and 9) also revealed a trend of increased activity on both substrates, as the intermodular linker length increased from no linkers at all to short 5-residue linkers, and from short to long linkers. Two-way analysis of variance (ANOVA) with interaction was used for statistical verification with length and the 14 scaffoldin arrangements as factors; no interaction was found for either substrate (*P* = 0.16 for Avicel; *P* = 0.0595 for pretreated wheat straw), indicating that linker length indeed had a significant effect on activity. The activities exhibited by the long, short and no-linker scaffoldins were thus observed to be significantly different from each other in the majority of the sets for both substrates. Of the 14 sets, 9 exhibited this general trend on Avicel, and 12 of the 14 sets on pretreated wheat straw.

Unlike the apparent dependence of activity on intermodular linker length of the scaffoldins, no clear trend could be discerned for position of the CBM or the cohesins (and consequent position of the respective enzymes used in this study). Designer cellulosomes based on scaffoldins having internal CBMs (Figures 8 and 9, top) exhibited similar levels of activity on the cellulosic substrates when compared to those based on scaffoldins with CBMs at their N or C termini (Figures 8 and 9, bottom). Likewise, the order of the cohesins in the scaffoldin and their nearest neighbor(s) did not have a perceptible effect on cellulolytic activity, using the three particular enzymes selected for this study.

## Discussion

The integration into a cellulosome system of cellulases and associated polysaccharide-degrading enzymes produced by anaerobic bacteria can facilitate their synergistic activity. However, it might also cause an opposite effect (anti-proximity) due to conformational restraint and steric clashes between the cellulases [59]. Our understanding of cellulosome architecture and its implications for cellulose hydrolysis is still limited due to the incredible heterogeneity of the cellulosome complex [25]. However, we are steadily gaining insight into the arrangement of the cellulosome complex and its functional consequences.

It appears that cellulosomal components exhibit diverse strategies that increase the plasticity of the complex and its interaction with its plant cell-wall polysaccharide substrates. This kind of flexibility may have a role in allowing enhanced access of the cellulases to the crystalline-cellulose substrate, which is interwoven together with hemicellulose, pectin and lignin components of the plant cell-wall. For example, the *C. thermocellum* type-I dockerin module displays internal sequence and structural symmetry that provide a dual mode of binding resulting in two different orientations of the dockerin-borne enzyme [60,61]. This type of plasticity would thus enable the enzyme to rapidly assume an alternative conformation for interaction with an untouched portion of its complex substrate.

In the past several years, selected studies have addressed the conformational flexibility of the cellulosome and its possible significance to biomass degradation. Early on, it was evident that isolated cellulosomes could attain an overall tight or loose conformation [1,62]. More recently [63], ultrastructural studies of a homogeneous mini-cellulosome containing three cohesin modules attached to three matching cellulases suggested that the flexibility of the linkers connecting consecutive cohesin modules could control structural transitions and thus regulate substrate recognition and degradation. In addition, the cellulases were found to be alternately projected from the scaffoldin in numerous directions, as was previously proposed based on the crystal structure of cohesins together with their adjacent linker segments [64,65]. This property of the scaffoldin can prevent steric clashes between neighboring catalytic modules.

The conformational events that occur upon integration of an enzyme into a cellulosome complex have been studied by examining the interaction between cellulosomal enzymes and cohesins by small-angle x-ray scattering (SAXS) [66,67]. Using this approach, the linker connecting the catalytic module of the *C. cellulolyticum* cellulosomal enzyme Cel48F to its dockerin was found to be extended and flexible when the enzyme is in its free form, but acquired a packed and rigid conformation upon binding to the matching cohesin. In another study [65], crystallographic evidence revealed the flexible nature of an extended intermodular linker of an *A. cellulolyticus* scaffoldin subunit. Combined SAXS and molecular dynamics studies revealed that the inter-cohesin linkers in binary and ternary complexes are intrinsically disordered, thereby resulting in extensive structural flexibility [66,68]. In addition, computational biology implementing a coarse-grained model was employed to study self-assembly and flexibility of cellulosomes [69]. In this work, the shape and modularity of the enzyme CbhA, was suggested to account for its tendency to bind more frequently to the scaffoldin due to its flexible nature and multimodularity that allowed a longer residence time around the scaffoldin.

The role of the intermodular linkers was further evaluated in a detailed study [70] wherein a cohesin dyad was employed for fabrication of hybrid cellulosomes with two model cellulases, Cel48F and Cel9G, derived from the mesophilic bacterium *C. cellulolyticum*. Several scaffoldin arrangements were used to form mini-cellulosome complexes, each of which contained two cohesins separated by various linker lengths, ranging from 4 to 128 residues, and various linker compositions, but without a CBM. All of the mini-complexes displayed similar activities on Avicel and PASC, and induced a similar 2-fold proximity between the two cellulases. Thus, in this case, the intermodular linker-length did not substantially affect the synergistic action of the two enzymes used in this study.

In the present study, we employed a synthetic biology (SynBio)-based strategy and strove to design a trivalent scaffoldin, which would serve as a basis for establishing a combinatorial library of scaffoldins. This library was then used for functional analysis of the position of the resident module and the length of the linkers between them. In doing so, we thus increased the level of complexity by adding a third cohesin, and hence a third cellulase, together with a supplementary CBM module that targets the scaffoldin to the substrate. This strategy would potentially allow 24 different modular arrangements of the scaffoldin. In addition to the native intermodular linkers, reduced linker length would enable examination of the effects of linker length on CBM-mediated targeting of the complex and enzyme proximity within the complex and their contribution to the overall deconstruction activity on cellulosic substrates. Owing to this broad design, we were able to overcome obstacles of cloning, expression and scaffoldin continuity, which limited the number of scaffoldins included in the final library. We thus succeeded in obtaining complete sets of scaffoldins, which contained all combinations of the desired positions of the modules and incremented lengths of their respective linker segments. Consequently, out of the 24 possible scaffoldin arrangements originally attempted, 14 complete sets were finally obtained, and their respective degradative capacity on cellulosic substrates was compared.

These 14 sets emulate the types of scaffoldin arrangements that occur in nature. For example, in simple cellulosome systems the CBM is positioned at the N terminus of the scaffoldin. We were thus able to express a complete set of six scaffoldins (scaffoldins 19 to 24 in Figure 3) that represent scaffoldins with an N-terminal CBM, with all of the possible permutations of the three cellulases. This scaffoldin set allowed us to determine if the position of a given enzyme in a trivalent designer cellulosome, relative to the other two, would appreciably influence the overall level of activity. An additional group of four scaffoldins carries the same arrangement of cohesins for all of the scaffoldins (*T, B*, *A*) with the CBM module at different positions in each of the scaffoldins: that is, a C-terminal position in scaffoldin 9, an internal position in scaffoldins 10 and 12, and N-terminal position in scaffoldin 22. The latter set allowed us to examine whether the position of the CBM would have a significant effect on the degradative capacity of the resultant designer cellulosome. Notably, the position of a C-terminal CBM has yet to be observed in a native multi-modular scaffoldin. The results indicated a lack of preferred scaffoldin arrangement using the specific *C. thermocellum* cellulases and cellulosic substrates described in this study - including the non-native scaffoldins (scaffoldin set 9, with the CBM at the C terminus) - which were fully functional in their contribution to degradation of cellulosic substrates. These results, however, do not necessarily indicate that other combinations of enzymes will not be affected by their location in a cellulosome. The combinatorial scaffoldin library can therefore be used as a platform to study the activities of other enzymes derived from different glycoside-hydrolase families or from different organisms, as well as other types of enzymes.

The results of the present study demonstrated that the use of native long intermodular linkers (average of 30.5 amino acids) between the scaffoldin-borne modules consistently resulted in higher cellulose-degrading activities. In addition, the designer cellulosome assemblies resulted in enhanced degradation of cellulose, due to the proximity between the enzymes and the targeting effect induced by the CBM3a module of the scaffoldin. The attachment of the CBM to the insoluble substrate may, however, restrict accessibility of the enzymes to the substrate due to attachment of the designer cellulosome on the surface of the cellulose [71]. In this context, the cohesin dyad-based hybrid-cellulosome [70] was free in the reaction solution and lacked the targeting function. Interestingly, in simple cellulose systems with short intermodular linkers, the CBM is positioned at the N terminus of the scaffoldin together with an *X*2 module, so the freedom of movement of enzymes attached to cohesins on the C-terminus of the scaffoldin is less compromised. In contrast, in complex cellulosome systems, with scaffoldins that are characterized by long intermodular linkers, the CBM module is typically positioned in an internal position in the polypeptide chain. The long linkers can thus compensate for the restricted movement induced by the CBM. The flexibility of the intermodular linker segments thus provides cooperativity among the cellulosomal glycoside hydrolases and results in synergy and increased cellulolytic activity [68]. Nevertheless, reduction of the intermodular linker length to 5-residues, or even their complete removal, failed to abolish the observed enhanced activity of the designer cellulosomes, thus underscoring the robust nature of cellulosome structure and architecture.

The different arrangements of the three enzymes on the designer cellulosome and the position of the CBM in the scaffoldin did not have a significant effect on the observed activity. In fact, we should consider that the designer cellulosomes are three-dimensional flexible complexes and not strings of cohesins bound to cellulases. What appears to be a different arrangement of cohesins in the designer scaffoldin would not necessarily result in a markedly different organization of the cellulases, owing to the flexibility or packing of the complex. Indeed, natural cellulosome complexes are intrinsically heterogeneous, since within a given species the cohesins of a given scaffoldin do not exhibit striking differences in specificity for the various dockerin-bearing enzymes, such that individual enzymes can bind to each of the scaffoldin-borne cohesin modules.

## Conclusions

A combinatorial library of a relatively simple designer-cellulosome system composed of three enzymes was prepared using a synthetic biology approach for examining the contribution of enzyme position, CBM location and intermodular linker length to cellulose-degrading activity. Longer intermodular scaffoldin linkers were shown to enhance the activity of the complex, whereas the position of the three particular enzymes selected for this study was of no apparent consequence. The high intrinsic flexibility of the intermodular linkers may be a major factor for refinement and optimization of the synergistic activity of the cellulosomal enzymes and may facilitate substrate targeting and binding of the scaffoldins, thereby maximizing the effectiveness of the cellulosome system as a cellulose-degrading machine. The scaffoldin library that we present in this paper can serve as a tool both to study the contribution of specified components of designer cellulosome assemblies and to explore unknown properties of cellulosome architecture, organization and action on plant-derived biomass.

## Materials and methods

### Cloning of cellulases

The recombinant wild-type family-48 exocellulase, Cel48S (48S-*t*)*,* was amplified from *C. thermocellum* ATCC 27405 genomic DNA with the following forward and reverse primers, 5′ CAGTCCATGGGTCCTACAAAGGCACCTAC 3′ and 5′ CGCGAAGCTTTTAATGGTGATGGTGATGGTGG 3′, respectively (*NcoI* and *HindIII* restriction sites in boldface), that allow their incorporation into pET28a. Similarly, the recombinant wild-type family-8 endocellulase, Cel8A (8A-*t*)*,* was cloned from the genomic DNA of *C. thermocellum* with the following forward and reverse primers, 5′ CAGTCCATGGGTGTGCCTTTTAACACAAA 3′ and 5′ CACGCTCGAGATAAGGTAGGTGGGGTATGC 3′ respectively, (*NcoI* and *XhoI* restriction sites in boldface). Likewise, the recombinant wild-type family-9 endocellulase, Cl9K (9K-*t*)*,* was amplified from the *C. thermocellum* genomic DNA and cloned into pET28a vector using the restriction-free (RF) method [46] with the following forward and reverse primers, 5′ GTTTAACTTTAAGAAGGAGATATACCATGGGCCATCACCATCACCATCAC*TTAGAAGACAAGTCTCCAAAGTTGCCGGAT* 3′ and 5′ GAGTGCGGCCGCAAGCTTGTCGACGGAGCTC*TTATTTATGTGGCAATACATCTATCTCTTTAAG* 3′ respectively, (gene-specific sequences are italicized, plasmid-specific sequences are shown in plain font, His-tag in bold). For the cloning of 9K-*a* with the divergent dockerin from *A. cellulolyticus*, the dockerin was amplified from the genomic DNA of *A. cellulolyticus* and used for the simultaneous insertion of the divergent dockerin and deletion of the wild-type dockerin into the wild-type 9K-*t* plasmid using the RF cloning method with the following forward and reverse primers, 5′ CTCGATGAAATTGACTTAATAACACCGCCAGGTACC*AAATTTATATATGGTGATGTTGATGGTAATG* 3′ and 5′ GAGTGCGGCCGCAAGCTTGTCGACGGAGCTC*TTATTCTTCTTTCTCTTCAACAGGGAATAAAAATATC* 3′ respectively (gene-specific sequences are italicized, plasmid-specific sequences are not). For the cloning of the chimaeric enzyme 8A-*b* with the *C. thermocellum* Cel8A catalytic module and a divergent dockerin from *B. cellulosolvens,* the catalytic module of Cel8A was amplified from *C. thermocellum* ATCC 27405 genomic DNA with the following forward and reverse primers, 5′ ATTCAACCATGGGTGTGCCTTTTAACACAAAATAC 3′ and 5′ ATATTGCTCGAGTAATGTGGTACCAATGAAGGTGTCGGATTCGACG 3′ respectively (*NcoI*, *KpnI* and *XhoI* restriction sites in boldface). The PCR product was cloned into a pET28a plasmid linearized with NcoI and XhoI restriction enzymes to yield p8A-CD. The dockerin was amplified from *B. cellulosolvens* genomic DNA with the following forward and reverse primers, 5′ ACTTTAGGTACCTCCAAAAGGCACAGCTAC 3′ and 5′ ATTAATCTCGAGCGCTTTTTGTTCTGCTGG 3′ respectively (*KpnI* and *XhoI* restriction sites in boldface). The resultant DNA was cloned into p8A-CD that was linearized with *KpnI* and *XhoI* to yield p8A-*b*.

### High-throughput computer-aided cloning of short- and no-linker scaffoldins

The core recursive construction step in this method required four basic enzymatic reactions: phosphorylation, elongation, PCR and Lambda exonucleation, and was performed as previously described by Linshiz et al. [44] using the primer sets listed in Additional file 5: Table S3.

The PCR product was amplified in order to yield sufficient amounts of DNA for subsequent cloning, by the following upper and lower primers, according to the modules that were located at the 5′ and 3′ of each scaffoldin construct: CtCohA2-Upper: 5′ CGCGAGCCATGGGGTCCGACGGTGTGGTAGTAG 3′, AcCohC3-Upper- 5′ CGCATGCCATGGGATCCGATTTACAGG 3′, CtScaACBM-Upper- 5′ CGACTCCCATGGCAAATACACCGGTATC 3′, BcCohB3-Upper- 5′ CGCAGGCCATGGGTAGTTCACCAGGAAATA 3′, CtCohA2-Lower- 5′ CGCACGCTCGAGTGTTGCATTGCCAACG 3′, AcCohC3-Lower: 5′ GGGCCGCTCGAGACTTGCAATTACCTC 3′, CtScaACBM-Lower- 5′ CTGTCGCTCGAGACTGCCACCGGGTTC 3′ and BcCohB3-Lower- 5′ GGACGGCTCGAGATTAGTTACAGTAATG 3′ (*NcoI* and *XhoI* restriction sites in boldface). The amplified product was digested by *NcoI* and *XhoI*, and ligated with *NcoI-XhoI* linearized pET28a vector (Novagene, Madison, WI, USA). Positive clones were selected by colony PCR and verified by sequencing. The approach was also attempted for production of long-linker scaffoldins with negative outcomes.

### Restriction-free (RF) cloning of long-linker scaffoldins

The trivalent long-linker scaffoldins were cloned in pET28a expression vector applying the RF method [46], using a simultaneous multi-component assembly approach. The assembly is described in detail in the Results section. Primer sets were designed for PCR amplification (Additional file 6: Table S4) and subsequent RF reactions were carried out using Phusion polymerase (Thermo Scientific, Hudson, NH, USA).

### Expression and purification of cellulases and designer scaffoldins

*Escherichia coli* BL21 (DE3) cells overproducing pET28a-scaffoldin genes or cellulases were grown at 37°C in Luria-Bertani broth supplemented with 50 μg/ml kanamycin (Sigma-Aldrich Chemical Co, St. Louis, MO, USA) to A_600_ = 0.8 to 1.0. The cultures were cooled to 16°C, and protein expression was induced by the addition of 0 to 1 mM isopropyl-1-thio-β-D-galactoside - IPTG (Fermentas UAB Vilnius, Lithuania), based on the results of predetermined optimization experiments. The cultures were incubated at 16°C for an additional 16 h, the cells were harvested by centrifugation (3500 g, 15 minutes), resuspendend in Tris-buffered saline (TBS, 137 mM NaCl, 2.7 mM KCl, 25 mM Tris–HCl, pH 7.4) supplemented with 5 mM imidazole (Merck KGaA, Darmstadt, Germany) and disrupted by sonication. The sonicate was heated for 20 minutes to 60°C and centrifuged (20,000 g, 30 minutes). The supernatant fluids were mixed with 4 ml of Ni-NTA beads for 1 h on a 20-ml Econo-pack column for batch purification at 4°C. The column was washed by gravity flow with 100 ml wash buffer (TBS, 50 mM imidazole) and elution was performed with 14 ml of elution buffer (TBS, 250 mM imidazole). For purification of the scaffoldins an additional affinity-purification step was applied: the eluted fractions were incubated in a 50-ml tube with 10 ml PASC (0.75 mg/ml) for 1 h at 4°C to allow binding of the CBM. The matrix was washed three times with TBS, containing 1 M NaCl, and three times with TBS without added salt. The scaffoldin was eluted with 1% triethylamine and neutralized with 1 M 2-(N-Morpholino) ethanesulfonic acid (MES) buffer pH 5. For both scaffoldin and cellulases the buffer was exchanged by dialysis against TBS, and the scaffoldin sample was concentrated using Amicon Ultra 15 ml 50,000 MWCO concentrators (Millipore, Bedford, MA, USA). Protein concentrations were estimated by the absorbance at 280 nm. The extinction coefficient was determined based on the known amino-acid composition of each protein using the ProtParam tool on the EXPASY server (http://www.expasy.org/tools/protparam.html) [72].

### Analysis of cohesin-dockerin specificity

The procedure of Barak et al. [73] was followed with minor modifications. Maxisorp ELISA plates (Nunc A/S, Roskilde, Denmark) were coated with 1 μg/ml each of the dockerin-containing enzymes 48S-*t*, 9K-*a* and 8A-*b*, and then interacted with 0.1 to 1,000 ng/μl of its matching CBM-cohesin (CBM-Coh *T*, CBM-Coh *A* and CBM-Coh *B*) counterpart. Rabbit-anti-CBM (diluted 1:3,000 in blocking buffer) was used as the primary antibody for detection of the interaction.

For analysis of the chimaeric scaffoldins, Maxisorp ELISA plates were coated with 1 μg/ml of the chimaeric scaffoldin and then interacted with 0.1 to 1,000 ng/μl of matching Xyn-Doc proteins which were prepared as described in Barak et al. [73]. These proteins are composed of xylanase T-6 from *Geobacillus stearothermophilus* fused to a dockerin module of appropriate specificity. Rabbit anti-xylanase T-6 antibody (diluted 1:10,000 in blocking buffer) was used as primary antibody for detection of the interaction. A secondary antibody preparation of goat-horseradish peroxidase (HRP)-labeled anti-rabbit antibody diluted 1:10,000 was added. The interaction was detected using TMB Substrate-Chromogen (Dako A/S, Glostrup, Denmark), and the reaction was terminated by the addition of 1 M sulfuric acid (H_2_SO_4_). Absorbance was measured at 450 nm.

### Non-denaturing PAGE

Equimolar concentrations of scaffoldins and matching enzymes (4 to 8 μg each protein) were mixed and added to similar volumes of interaction buffer (TBS with 10 mM calcium chloride (CaCl_2_) and 0.05% Tween20). Double-distilled water (DDW) was added to a final volume of 30 μl. The proteins were incubated at 37°C for 2 h to allow complex formation. Non-denaturing sample buffer (192 mM glycine, 25 mM Tris) was added, and a total of 15 μl/lane was subjected to PAGE (7.5 to 9.0% acrylamide gels), using a Bio-Rad Laboratories (Hercules, CA, USA) power pack 300. Single components (scaffoldin and enzymes) were used as markers. The remaining 15 μl were used for analysis on SDS-PAGE.

### Size-exclusion high performance liquid chromatography (HPLC)

Equimolar protein concentrations (450 picomoles scaffoldin or enzyme) were diluted in 300 μl of loading buffer (TBS, pH 7.4), supplemented with 2 mM of CaCl_2_. For the formation of designer cellulosome complexes, equimolar concentrations of a scaffoldin and enzymes were incubated at 37°C for 2 h with similar volumes of interaction buffer (TBS with 10 mM CaCl_2_ and 0.05% Tween20), and loading buffer was added to a final volume of 300 μl. The reactions were injected onto an analytical Superdex 200 HR 10/30 column using an AKTA fast-performance liquid chromatography system (GE Healthcare, Uppsula, Sweden) and loading buffer at a flow rate of 0.5 ml · min^-1^. Eluted proteins were detected at 280 nm and fractions (0.5 ml) concentrated and analyzed using SDS-PAGE gels.

### Preparation of cellulose-enriched (pretreated) wheat straw

Wheat-straw sections were cut with shears into pieces 2 to 5 cm and subsequently ground with a knife mill (Waring Products Inc., Torrington, CT, USA) at 15,000 rpm for 1 minute. The resultant powder was passed through a sieve with mesh =10 to obtain a final biomass powder having an average particle size of 1 to 3 mm. A sample (20 g) of the resultant powder was treated with 85 ml of 5% (v/v) nitric acid for 1 h at 115°C. The acid-treated biomass was washed with DDW and treated further with 150 ml of 1.5% v/v sodium hydroxide (NaOH) for 1 h at 100°C and washed with DDW, yielding a cellulose-enriched substrate.

### Determination of wheat-straw substrate chemical composition

The chemical composition of the samples was determined according to the following improvement of the Technical Association of the Pulp and Paper Industry (TAPPI)-method [74]. For hemicellulose content, samples were boiled with 2% HCl for 2 h, washed with DDW and ethanol and dried at 105°C to constant weight (about 2 to 3 h). For cellulose content, samples were boiled with an ethanolic nitric acid (HNO_3_) solution for 1 h, washed with DDW and ethanol, and dried at 105°C to constant weight (about 2 to 3 h). For lignin content, samples were swollen in 72% H_2_SO_4_ at room temperature for 2 h, diluted with DDW to 8 to 10% acid, hydrolyzed with boiling diluted H_2_SO_4_ (8 to 10%) for 2 h, washed with DDW and ethanol, and dried at 105°C to constant weight (about 2 to 3 h). Total solid content was determined by drying the samples at 105°C for 2 h.

### Activity assays

The hydrolysis reactions were carried out in a total volume of 200 μl, and consisted of reaction buffer (100 mM sodium acetate buffer pH 5.5, 24 mM CaCl_2_, 4 mM ethylenediaminetetraacetic acid (EDTA)), 0.5 μM of each protein and 2% w/v Avicel (Sigma-Aldrich) or 3.5 g/L pretreated (cellulose-enriched) wheat straw. Prior to the addition of the substrate, each scaffoldin was incubated with equimolar quantities of the three enzymes for 2 h at 37°C with a similar volume of interaction buffer (TBS with 10 mM CaCl_2_ and 0.05% Tween 20). The reaction was carried out for 24 to 72 h (Avicel) or 3 to 24 h (pretreated wheat straw) at 50°C and terminated by immersion in ice water. The substrate was pelleted by centrifugation at maximum speed (20,800 × g, 10 to 15 minutes), and 100 μl of the supernatant was transferred to a new tube. Dinitrosalycylic acid (DNS, 150 μl) was added, and the samples were boiled for 10 minutes. The absorbance was measured at 540 nm and the reducing sugars were determined according to a glucose calibration curve. Each assay was repeated three times in triplicate.

## Abbreviations

Bp: base pairs; CBM and c: the family 3a carbohydrate binding module of the CipA scaffoldin from *C. thermocellum*; 8A-b: chimaeric enzyme consisting of the catalytic module of Cel8A from *C. thermocellum* fused to a divergent dockerin from *B. cellulosolvens*; 9K-a: chimaeric enzyme consisting of the catalytic module of Cel9K from *C. thermocellum* fused to a divergent dockerin from *A. cellulolyticus*; 48S-t: recombinant wild-type cellulosomal enzyme from *C. thermocellum* consisting of the catalytic module and dockerin; Coh A: *A. cellulolyticus* cohesin C3 module fused to CBM3a from *C. thermocellum* CipA scaffoldin; Coh B: *B. cellulosolvens* cohesin B3 module fused to CBM3a from *C. thermocellum* CipA scaffoldin; Coh T: *C. thermocellum* cohesin A2 module fused to CBM3a from *C. thermocellum* CipA scaffoldin; no linkers: Scaffoldin without intermodular linkers; short linkers: Scaffoldin with short intermodular linkers; long linkers: Scaffoldin with long intermodular linkers; DDW: double-distilled water; DNS: dinitrosalycylic acid; ELISA: enzyme-linked immunosorbent assay; NTA: Ni-nitrilotriacetic acid; PASC: phosphoric acid-swollen cellulose; PCR: polymerase chain reaction; RF: restriction-free; Scaf4N: Scaffoldin number 4 without intermodular linkers (the numbers in the terminology change according to the given scaffoldin set); Scaf4S: Scaffoldin number 4 with short intermodular linkers; Scaf4L: Scaffoldin number 4 with long intermodular linkers; TBS: Tris-buffered saline; Xyn-Doc: xylanase T-6 from *Geobacillus stearothermophilus* fused to a dockerin module of an appropriate specificity.

## Competing interests

The authors declare that they have no competing interests.

## Authors’ contributions

YV and MS designed and carried out the experiments. YV analyzed results and wrote the manuscript. TU and YP designed primers and cloned the long-linker scaffoldin. ES TBY and YM designed the primers and produced the DNA of the no-linker and short-linker scaffoldins for subsequent cloning. YB, RL and EAB assisted in the experimental design and reviewed the manuscript. All authors read and approved the manuscript.

## Supplementary Material

Additional file 1: Table S1 Molecular weights of the different chimaeric scaffoldins produced in this work. Click here for file

Additional file 2: Figure S1 Demonstration of cellulose-binding ability of cellulases and scaffoldin used in this work. Cellulose-binding assays were performed as described earlier [30]. The enzymes and scaffoldin (5–20 mg) were incubated with Avicel, the suspension was centrifuged, and the supernatant fluids with the unbound fraction (S) and pellet with the bound fraction (P) were subjected to SDS-PAGE analysis. Left: Cel48S-*t* (lane 1 and 2), Cel9K-*a* (lane 3 and 4), Cel8A-*b* (lane 5 and 6), and the positive control scaffoldin 17L bearing a CBM3a module (lane 7 and 8). Mw – Molecular weight markers (kDa). Click here for file

Additional file 3: Figure S2 SDS-PAGE analysis of the 42 scaffoldins in the final scaffoldin library. Each scaffoldin (2.5 to 3.0 μg) was subjected to 10% SDS-PAGE. The modular composition of each set and the scaffoldin number is denoted at the bottom of each gel (as described in Figure 3 of the main text of the article). Scaffoldins His-10L and His-9L refer to Scaf10L and Scaf9L in the manuscript and were modified so that the histidine-tag was transferred to the N terminus of the scaffoldin. The migration pattern of an additional control scaffoldin, No CBM (Scaf20ΔCBM), is also shown. All of the scaffoldins display a major band corresponding to their calculated molecular weights (Table S2). Mw, molecular mass marker. Click here for file

Additional file 4: Table S2 Enzymatic activity measured as reducing sugars (mM) released by the various enzyme combinations used in this study when tested on A) Avicel after 24, 48 and 72 h and tested on B) pretreated wheat straw after 3, 6 and 24 h. All reactions were carried out in triplicate. Standard deviations from three separate experiments are indicated. Click here for file

Additional file 5: Table S3 Primer sequences used for the cloning of the no inter-modular linker and short inter-modular linker scaffoldins. Click here for file

Additional file 6: Table S4 Primer sequences used for the cloning of the long inter-modular linker scaffoldins. Modules are color-coded: plasmid sequences (red), Coh *T* (black), carbohydrate binding module (CBM) (*c*)(brown), Coh *B* (green), Coh *A* (magenta). Primer sequences that appear in boldface are primers that already appear in the list in the primer set of a former scaffoldin. Click here for file
