# Supplementary material for: A synthetic biology approach for evaluating the functional contribution of designer cellulosome components to deconstruction of cellulosic substrates
Source: Biotechnol Biofuels. 2013 Dec 16;6:182. doi: 10.1186/1754-6834-6-182 (PMC3878649; doi:10.1186/1754-6834-6-182)
Supplement: Additional file 1: Table S1 — Molecular weights of the different chimaeric scaffoldins produced in this work. [file 1754-6834-6-182-S1.docx]

| **Scaffoldin number** | **Modular composition** | | | | **Molecular weight (Da)** | | |
| --- | --- | --- | --- | --- | --- | --- | --- |
|  |  |  |  |  | **No  linkers** | **Short linkers** | **Long linkers** |
| **20** | *c* | A | B | T | 66,046 | 67,569 | 74,693 |
| **19** | *c* | *A* | *T* | *B* | 66,046 | 67,509 | 75,363 |
| **23** | *c* | *B* | *A* | *T* | 66,046 | 67,569 | 74,693 |
| **24** | *c* | *B* | *T* | *A* | 66,046 | 67,499 | 75,362 |
| **21** | *c* | *T* | *A* | *B* | 66,046 | 67,509 | 75,363 |
| **22** | *c* | *T* | *B* | *A* | 66,046 | 67,499 | 75,362 |
| **6** | *A* | *c* | *B* | *T* | 66,046 | 67,569 | 74,693 |
| **5** | *A* | *c* | *T* | *B* | 66,046 | 67,509 | 75,363 |
| **17** | *B* | *c* | *A* | *T* | 66,046 | - | 74,693 |
| **18** | *B* | *c* | *T* | *A* | 66,046 | 67,499 | 75,362 |
| **11** | *T* | *c* | *A* | *B* | 66,046 | 67,509 | 75,363 |
| **12** | *T* | *c* | B | *A* | 66,046 | 67,499 | 75,362 |
| **4** | *A* | *B* | *c* | *T* | 66,046 | 67,569 | 74,693 |
| **2** | *A* | *T* | *c* | *B* | - | - | - |
| **14** | *B* | *A* | *c* | *T* | - | - | - |
| **16** | *B* | *T* | *c* | *A* | - | - | - |
| **8** | *T* | *A* | *c* | *B* | - | - | - |
| **10** | *T* | *B* | *c* | *A* | 66,046 | 67,499 | 75,362 |
| **3** | *A* | *B* | *T* | *c* | - | 67,597 | - |
| **1** | *A* | *T* | *B* | *c* | - | - | - |
| **13** | *B* | *A* | *T* | *c* | - | - | - |
| **15** | *B* | *T* | *A* | *c* | - | - | - |
| **7** | *T* | *A* | *B* | *c* | - | - | - |
| **9** | *T* | *B* | *A* | *c* | 66,046 | 67,597 | 75,090 |
