# Supplementary figures and images for: A synthetic biology approach for evaluating the functional contribution of designer cellulosome components to deconstruction of cellulosic substrates
Source: Biotechnol Biofuels. 2013 Dec 16;6:182. doi: 10.1186/1754-6834-6-182 (PMC3878649; doi:10.1186/1754-6834-6-182)

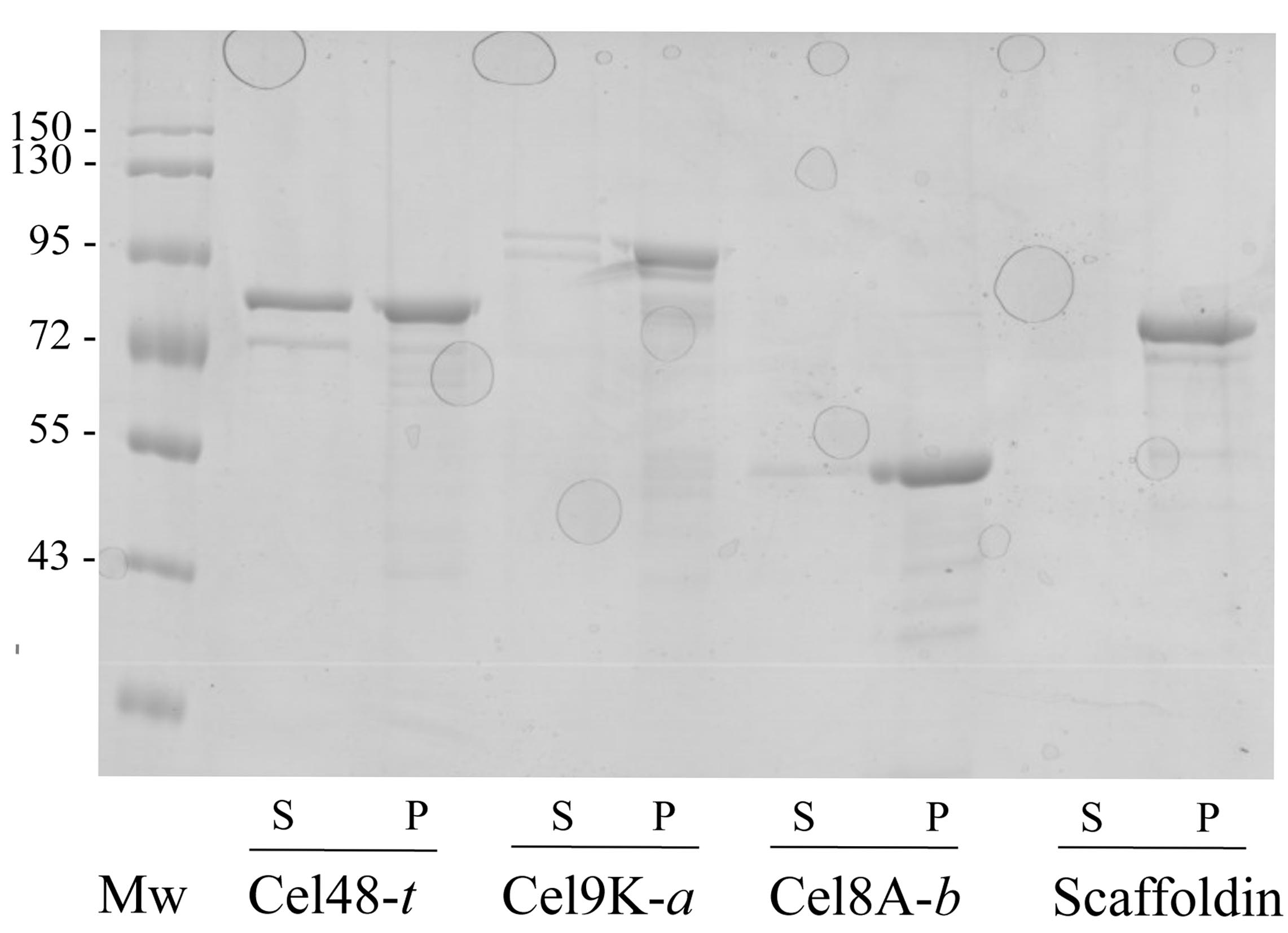

Supplement: Additional file 2: Figure S1 — Demonstration of cellulose-binding ability of cellulases and scaffoldin used in this work. Cellulose-binding assays were performed as described earlier [30]. The enzymes and scaffoldin (5–20 mg) were incubated with Avicel, the suspension was centrifuged, and the supernatant fluids with the unbound fraction (S) and pellet with the bound fraction (P) were subjected to SDS-PAGE analysis. Left: Cel48S-t (lane 1 and 2), Cel9K-a (lane 3 and 4), Cel8A-b (lane 5 and 6), and the positive control scaffoldin 17L bearing a CBM3a module (lane 7 and 8). Mw – Molecular weight markers (kDa). [file 1754-6834-6-182-S2.jpeg]

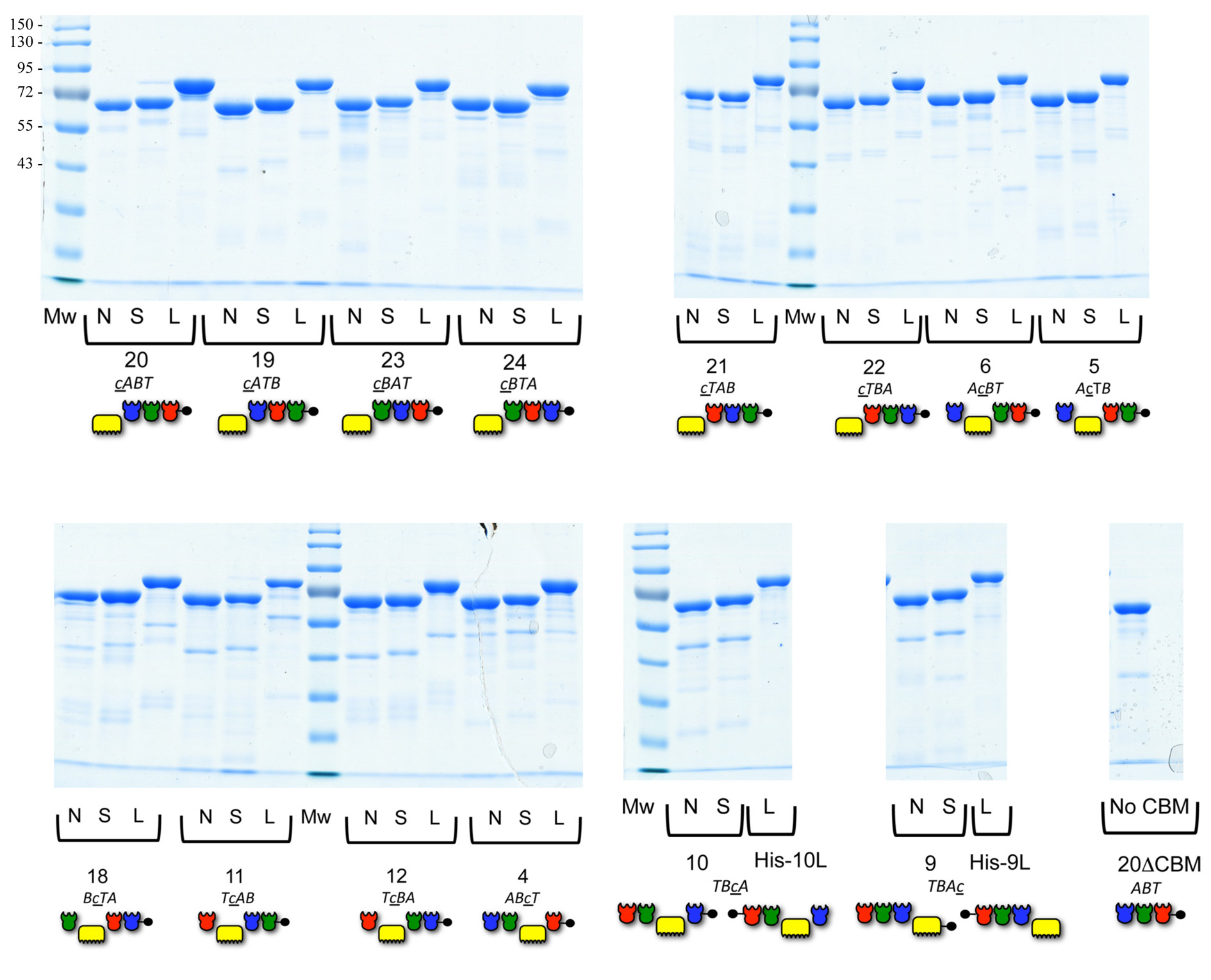

Supplement: Additional file 3: Figure S2 — SDS-PAGE analysis of the 42 scaffoldins in the final scaffoldin library. Each scaffoldin (2.5 to 3.0 μg) was subjected to 10% SDS-PAGE. The modular composition of each set and the scaffoldin number is denoted at the bottom of each gel (as described in Figure 3 of the main text of the article). Scaffoldins His-10L and His-9L refer to Scaf10L and Scaf9L in the manuscript and were modified so that the histidine-tag was transferred to the N terminus of the scaffoldin. The migration pattern of an additional control scaffoldin, No CBM (Scaf20ΔCBM), is also shown. All of the scaffoldins display a major band corresponding to their calculated molecular weights (Table S2). Mw, molecular mass marker. [file 1754-6834-6-182-S3.jpeg]
