# Supplementary material for: A synthetic biology approach for evaluating the functional contribution of designer cellulosome components to deconstruction of cellulosic substrates
Source: Biotechnol Biofuels. 2013 Dec 16;6:182. doi: 10.1186/1754-6834-6-182 (PMC3878649; doi:10.1186/1754-6834-6-182)
Supplement: Additional file 4: Table S2 — Enzymatic activity measured as reducing sugars (mM) released by the various enzyme combinations used in this study when tested on A) Avicel after 24, 48 and 72 h and tested on B) pretreated wheat straw after 3, 6 and 24 h. All reactions were carried out in triplicate. Standard deviations from three separate experiments are indicated. [file 1754-6834-6-182-S4.docx]

**A) Avicel:**

|  |  | **24 h** | **48 h** | **72 h** |
| --- | --- | --- | --- | --- |
|  | **(1) 48S-*t*** | - | - | 0.83 ± 0.05 |
|  | **(2) 9K-*a*** | - | - | 2.46 ± 0.6 |
|  | **(3) 8A-*b*** | - | - | 1.63 ± 0.03 |
| **A** | **Sum of (1)+(2)+(3)** | - | - | 4.92 |
| **B** | **Free** | 3.47 ± 0.36 | 4.13 ± 0.22 | 4.79 ± 0.14 |
| **C** | **No CBM** | - | - | 5.6 ± 0.42 |
| **D** | **Scaf21L** | 6.23 ± 0.38 | 6.88 ± 0.32 | 8.74 ± 0.12 |
| **(B/A)** | **Observed/Calculated** | - | - | 0.97 |
| **(C/B)** | **Proximity effect** | - | - | 1.17 |
| **(D/C)** | **Targeting effect** | - | - | 1.56 |
| **(D/B)** | **Overall enhancement** | 1.79 | 1.66 | 1.82 |

**B) Pretreated wheat straw:**

|  |  | **3 h** | **6 h** | **24 h** |
| --- | --- | --- | --- | --- |
|  | **(1) 48S-*t*** | 0.03 ± 0.03 | - | 0.02 ± 0.01 |
|  | **(2) 9K-*a*** | 0.45 ± 0.07 | - | 1.71 ± 0.52 |
|  | **(3) 8A-*b*** | 0.04 ±0.02 | - | 0.09 ± 0.05 |
| **A** | **Sum of (1)+(2)+(3)** | 0.52 | - | 1.82 |
| **B** | **Free** | 1.90 | 2.57 ± 0.27 | 3.89 ± 0.50 |
| **C** | **No CBM** | 2.12 ± 0.13 | 2.95 ± 0.14 | 4.43 ± 0.69 |
| **D** | **Scaf21L** | 3.67 ± 0.21 | 4.92 ± 0.68 | 7.53 ± 0.53 |
| **(B/A)** | **Observed/Calculated** | 3.65 | - | 2.14 |
| **(C/B)** | **Proximity effect** | 1.12 | 1.15 | 1.14 |
| **(D/C)** | **Targeting effect** | 1.73 | 1.67 | 1.70 |
| **(D/B)** | **Overall enhancement** | 1.93 | 1.91 | 1.93 |
