# Supplementary material for: A synthetic biology approach for evaluating the functional contribution of designer cellulosome components to deconstruction of cellulosic substrates
Source: Biotechnol Biofuels. 2013 Dec 16;6:182. doi: 10.1186/1754-6834-6-182 (PMC3878649; doi:10.1186/1754-6834-6-182)
Supplement: Additional file 5: Table S3 — Primer sequences used for the cloning of the no inter-modular linker and short inter-modular linker scaffoldins. [file 1754-6834-6-182-S5.docx]

| **Name** | **Sequence** | **Length** |
| --- | --- | --- |
| EBCell2_P1 | CCATGGGGTCAGACGGTGTGGT | 22bp |
| EBCell2_P2 | CTCGAGATTAGTTACAGTAATGC | 23bp |
| EBCell2_P3 | ACTGCCACCGGGTTCTTTACCCCATACAAG | 30bp |
| EBCell2_P4 | CGGGTTCTTTACCCCATACAAG | 22bp |
| EBCell2_P5 | AAATCGGATCCTGTTGCATTGCCAACGTTAA | 31bp |
| EBCell2_P6 | GCAATGCAACAGGATCCGATTTACAGGTTGACA | 33bp |
| EBCell2_P7 | TGTTGCATTGCCAACGTTAA | 20bp |
| EBCell2_P8 | CCATGGGGTCAGACGGTGTGGTAGTAGA | 28bp |
| EBCell2_P9 | GGATCCGATTTACAGGTTGACA | 22bp |
| EBCell2_P10 | ACCTCAATTTTTCCATCATTGTAAACAACATTTTTGATT | 39bp |
| EBCell2_P11 | ATGTTGTTTACAATGATGGAAAAATTGAGGTAATTGCAAGTGC | 43bp |
| EBCell2_P12 | AAAATTGAGGTAATTGCAAGTGC | 23bp |
| EBCell2_P13 | CTTCAGATACTACTAACTCAATCAATCCTCAGTTCAAGGTT | 41bp |
| EBCell2_O15 | AAAATTGAGGTAATTGCAAGTGCAAATACACCGGTATCAGG | 41bp |
| EBCell2_P16 | CTCGAGATTAGTTACAGTAATGCTTCCATCT | 31bp |
| EBCell2_P17 | GGATTGATTGAGTTAGTAGTATCTGAAGGATTGCTGTTGTAG | 42bp |
| EBCell2_P18 | GGGTAAAGAACCCGGTGGCAGTAGTTCACCAGGAAATAAAATGAAAAT | 48bp |
| EBCell2_P19 | CCATGGGTAGTTCACCAGGA | 20bp |
| EBCell2_P20 | CTCGAGTGTTGCATTGCC | 18bp |
| EBCell2_P21 | ATTACTGTAACTAATGGATCCGATTTACAGGTTGACATTGGA | 42bp |
| EBCell2_P22 | CGATTTACAGGTTGACATTGGA | 22bp |
| EBCell2_P23 | GTCTGAACTGCCACCGGGTTCTTTA | 25bp |
| EBCell2_P24 | AGAACCCGGTGGCAGTTCAGACGGTGTGGTAGTAGA | 36bp |
| EBCell2_P25 | TCAGACGGTGTGGTAGTAGA | 20bp |
| EBCell2_P26 | CTCGAGTGTTGCATTGCCAACGTTAA | 26bp |
| EBCell2_P27 | ACCTGTAAATCGGATCCATTAGTTACAGTAATGCTTCCATCT | 42bp |
| EBCell2_P28 | CTCGAGACTGCCACCGGGTT | 20bp |
| EBCell2_P29 | CTGAATTAGTTACAGTAATGCTTCCATCT | 29bp |
| EBCell2_P30 | TGGAAGCATTACTGTAACTAATTCAGACGGTGTGGTAGTAGA | 42bp |
| EBCell2_P31 | CTCGAGACTGCCACCG | 16bp |
| EBCell2_P32 | CCATGGGATCCGATTTACAGG | 21bp |
| EBCell2_P33 | TTGGTCGGCGTATTAGTTACAGTAATGCTTCCATC | 35bp |
| EBCell2_P34 | GTATTAGTTACAGTAATGCTTCCATC | 26bp |
| EBCell2_P35 | CCCCGACGCAATCAGACGGTGTGGTAGTAGA | 31bp |
| EBCell2_P36 | TTGCGTCGGGGTAGGACTTGCAATTACCTCAATTTTTC | 38bp |
| EBCell2_P37 | CCGACGAAAGGGGCAAGTTCACCAGGAAATAAAATG | 36bp |
| EBCell2_P38 | GTCGGTGTTGCATTGCCA | 18bp |
| EBCell2_P39 | TCAGACGGTGTGGTAGTAGAAATTGG | 26bp |
| EBCell2_P40 | GGTGAACTTGCCCCTTTCGTCGGTGTTGCATTGCCA | 36bp |
| EBCell2_P41 | ACACCGTCTGATTGCGTCGGGGTAGGACTTGCAATTACCTCAATTTTTC | 49bp |
| EBCell2_P42 | CATTACTGTAACTAATACGCCGACCAATACTGCAAATACACCGGTATCAGGC | 52bp |
| EBCell2_P43 | TGCTCGGAACAACACTGCCACCGGGTTC | 28bp |
| EBCell2_P44 | ACACTGCCACCGGGTTC | 17bp |
| EBCell2_P45 | TGCTGCCCCTTTCGTCGGTGTTGCATTGCCAA | 32bp |
| EBCell2_P46 | GTCGGTGTTGCATTGCCAA | 19bp |
| EBCell2_P47 | CCCCGACGCAATCAGACGGTGTGGTAGTAGAAATTGG | 37bp |
| EBCell2_P48 | CAACACCGACGAAAGGGGCAGCAAATACACCGGTATCA | 38bp |
| EBCell2_P49 | GGTGGCAGTGTTGTTCCGAGCACGAGTTCACCAGGAAATAAAATGAAAAT | 50bp |
| EBCell2_P50 | CCGACCAATACTGGATCCGATTTACAGGTTGACA | 34bp |
| EBCell2_P51 | TAGTAACCTTGAACTGAGGATTGATTGAGTTAGTAG | 36bp |
| EBCell2_P52 | CAATCAATCCTCAGTTCAAGGTTACTAATACCGGAAGC | 38bp |
| EBCell2_P53 | GGCAGCAAATACACCGGTATC | 21bp |
| EBCell2_P54 | CAACACCGACGAAAGGGGCAGCAAATACACCGGTA | 35bp |
| EBCell2_P55 | AAATCGGATCCAGTATTGGTCGGCGTATTAGTTACAGTAATGCTTCCATC | 50bp |
| EBCell2_P56 | CGGCGTATTAGTTACAGTAATGCTTCCATCTTTAAAT | 37bp |
| EBCell2_P57 | GCATTACTGTAACTAATACGCCGACCAATACTG | 33bp |
| EBCell2_P58 | CTAATACGCCGACCAATACTG | 21bp |
| EBCell2_P59 | TCGGGGTAGGACTTGCAATTACCTCAATTTTTCCA | 35bp |
| EBCell2_P60 | CTTGCAATTACCTCAATTTTTCCA | 24bp |
| EBCell2_P61 | GAACAACACTGCCACCGGGTTCTTTACCC | 29bp |
| EBCell2_P62 | GCCACCGGGTTCTTTACCC | 19bp |
| EBCell2_P63 | CTAATACGCCGACCAATACTGCAAATACACCG | 32bp |
| EBCell2_P64 | AGTAACCTTGAACTGAGGATTGATTGAGTTAGTAG | 35bp |
| EBCell2_P65 | GACCAATACTGCAAATACACCGGTATCAGGC | 31bp |
| EBCell2_P66 | GAACCCGGTGGCAGTGTTGTTCCGAGCACGGGATCCGATTTACAGGTTGA | 50bp |
| EBCell2_P67 | GTAATTGCAAGTCCTACCCCGACGCAATCAGACGGTGTGGTAGTAGAA | 48bp |
| EBCell2_P68 | CCATGGCAAATACACCGGT | 19bp |
| EBCell2_P69 | GCGTCGGGGTAGGACTTGCAATTACCTCAATTTTTC | 36bp |
| EBCell2_P70 | TAATTGCAAGTCCTACCCCGACGCAATCAG | 30bp |
| EBCell2_P71 | CCATGGCAAATACACCGGTA | 20bp |
| EBCell2_P72 | GGACTTGCAATTACCTCAATTTTTC | 25bp |
| EBCell2_P73 | TACCCCGACGCAATCAG | 17bp |
| EBCell2_P74 | CTGGTGAACTTGCCCCTTTCGTCGGTG | 27bp |
| EBCell2_P75 | ACGAAAGGGGCAAGTTCACCAGGAAATAAAATGAAAATT | 39bp |
| EBCell2_P76 | TACCCCGACGCAATCAGACGGTGTGGTAGTAGAAATTGG | 39bp |
| EBCell2_P77 | GACGGTGTGGTAGTAGAAATTGG | 23bp |
| EBCell2_P78 | GGTGGCAGTGTTGTTCCGAGCACGGGATCCGATTTACAGGTTGA | 44bp |
| EBCell2_P79 | GTTGTTCCGAGCACGAGTTCACCAGGAAATAAAATGAAAATTC | 43bp |
| EBCell2_P80 | GAGTTCACCAGGAAATAAAATGAAAATTC | 29bp |
| EBCell2_P81 | CCCCGACGCAATCAGACGGTGTGGTAGTAGAA | 32bp |
| EBCell2_P82 | TGGTGAACTCGTGCTCGGAACAACACTGCCACCGGGTTC | 39bp |
| EBCell2_P83 | CTCGAGACTGCCACCGGGTTCTTTA | 25bp |
| EBCell2_P84 | CATTACTGTAACTAATACGCCGACCAATACTATCAGTG | 38bp |
| EBCell2_P85 | TTGCCCCTTTCGTCGGTGTTGCATTGCCA | 29bp |
| EBCell2_P86 | GCAACACCGACGAAAGGGGCAACACCAACCA | 31bp |
| EBCell2_P87 | CGTCGGGGTAGGACTTGCAATTACCTCAATTTTTC | 35bp |
| EBCell2_P88 | AATTGCAAGTCCTACCCCGACGCAATCAG | 29bp |
| EBCell2_P89 | GTCGGTGTTGCATTGCCAACGTTAA | 25bp |
| EBCell2_P90 | GCCAATTTCTACTACCACACCGTCTGATGG | 30bp |
| EBCell2_P91 | TACCCCGACGCAATCAGCCACTCCAACGGTAACTCCTTCAGCCACCGCGACGCCTACC | 58bp |
| EBCell2_P93 | GAAAGGGGCAACACCAACCA | 20bp |
| EBCell2_P94 | CTGGTGAACTCGTTGGGGTATTAGTTGGCACTG | 33bp |
| EBCell2_P95 | AATACCCCAACGAGTTCACCAGGAAATAAAATGAAAAT | 38bp |
| EBCell2_P96 | GCCGACCAATACTATCAGTG | 20bp |
| EBCell2_P98 | CCAAAGCCAAACCCGTTAG | 19bp |
| EBCell2_P99 | CCAAAGCCAAACCCGTTAGCAAATACACCGGTATCAGG | 38bp |
| EBCell2_P100 | GGTGGCAGTGTTGTTCCGAGCACGCA | 26bp |
| EBCell2_P101 | TGTTGCCCCTTTCGTCGGTGTTGCATTGCCA | 31bp |
| EBCell2_P102 | CAACACCGACGAAAGGGGCAACACCAACCAAC | 32bp |
| EBCell2_P103 | GAAAGGGGCAACACCAACCAAC | 22bp |
| EBCell2_P104 | TGTATTTGCCGTTGGGGTATTAGTTGGCACTG | 32bp |
| EBCell2_P105 | ACTAATACCCCAACGGCAAATACACCGGTATCAGG | 35bp |
| EBCell2_P106 | TGTTCCGAGCACGCA | 15bp |
| EBCell2_P108 | GATCCCTCCTAGCAGTTCACCAGGAAATAAAATGAAAATTC | 41bp |
| EBCell2_P109 | ACCACACCGTCTGATAACGGGTTTGGCTTTGG | 32bp |
| EBCell2_P110 | ACCCGTTATCAGACGGTGTGGTAGTAGAAATTGG | 34bp |
| EBCell2_P111 | TGATAACGGGTTTGGCTTTGG | 21bp |
| EBCell2_P112 | TTATTTCCTGGTGAACTTGGCGTTACAGTCG | 31bp |
| EBCell2_P113 | CGCCAAGTTCACCAGGAAATAAAATGAAAATTCAAATTG | 39bp |
| EBCell2_P114 | TACCCCGACGCAATCAGCCACTCCAACGGTAACTCCTTCAGCCACCGCGACGC | 53bp |
| EBCell2_P115 | GAACTTGGCGTTACAGTCGGCGTAGCACTCTGGGTAGGCGTCGCGGTGGCTGAAGG | 56bp |
| EBCell2_P116 | GGTGTATTTGCCGTTGGGGTATTAGTTGGCACTG | 34bp |
| EBCell2_P117 | TAATACCCCAACGGCAAATACACCGGTATCAGG | 33bp |
| EBCell2_P118 | GTTGGGGTATTAGTTGGCACTG | 22bp |
| EBCell2_P119 | CGGTGTATTTGCTAACGGGTTTGGCTTTGGCGT | 33bp |
| EBCell2_P120 | AAACCCGTTAGCAAATACACCGGTATCAGG | 30bp |
| EBCell2_P121 | AACGGGTTTGGCTTTGGCGT | 20bp |
| EBCell2_P122 | AACGGGTTTGGCTTTGGCG | 19bp |
| EBCell2_P123 | AGCAAATACACCGGTATCAGG | 21bp |
| EBCell2_P125 | CCCTCCTAGCTCAGACGGTGTGGTAGTAGA | 30bp |
| EBCell2_P126 | GGTGGCAGTGTTGTTCCGAGCACGCAGC | 28bp |
| EBCell2_P127 | CTGATACCGGTGTATTTGCTGGCGTTACAG | 30bp |
| EBCell2_P128 | CCAGCAAATACACCGGTATCAGGCAATTTGAAG | 33bp |
| EBCell2_P129 | TACCCCGACGCAATCAGCCACTCCAACGGTAACTCCTTCAGCCACCGCGACGCCT | 55bp |
| EBCell2_P130 | TGTATTTGCTGGCGTTACAGTCGGCGTAGCACTCTGGGTAGGCGTCGCGGTGGCTGAAGG | 60bp |
| EBCell2_P131 | TGTTCCGAGCACGCAGC | 17bp |
| EBCell2_P132 | TATTGGTCGGCGTATTAGTTACAGTAATGCTTCCATC | 37bp |
| EBCell2_P133 | TTACTGTAACTAATACGCCGACCAATACTATCAGTG | 36bp |
| EBCell2_P134 | TTTCTACTACCACACCGTCTGATAACGGGT | 30bp |
| EBCell2_P135 | TTATCAGACGGTGTGGTAGTAGAAATTGGCAAAGTTACG | 39bp |
| EBCell2_P136 | GCCGACCAATACTATCAGTGTTACTCCGACAAACAATTCGACTCCTACGAATA | 53bp |
| EBCell2_P137 | ACACCGTCTGATAACGGGTTTGGCTTTGGCGTACTGTTATTCGTAGGAGTCGA | 53bp |
| EBCell2_P138 | CCATGGGGTCAGACGGTGTGGTAGTAGAAATTGG | 34bp |
| EBCell2_P139 | CCATGGGGTCAGACGGTG | 18bp |
| EBCell2_P140 | TTGCGTCGGGGTAGGACTTGCAATTACCTCAATTT | 35bp |
| EBCell2_P141 | TGCAAGTCCTACCCCGACGCAATCAGC | 27bp |
| EBCell2_P142 | TAGGACTTGCAATTACCTCAATTTTTC | 27bp |
| EBCell2_P143 | CCTGTAAATCGGATCCCGTTGGGGTATTAGTTG | 33bp |
| EBCell2_P144 | CCAACGGGATCCGATTTACAGGTTGACATTGGA | 33bp |
| EBCell2_P145 | GAAAGGGGCAACACCAACCAACACAGCCACGCCAACAAAGTCGGCAACCGCGACCC | 56bp |
| EBCell2_P146 | GATCCCGTTGGGGTATTAGTTGGCACTGAAGGGCGTGTCGGGGTCGCGGTTGCCGA | 56bp |
| EBCell2_P147 | CCCCGACGCAATCAGC | 16bp |
| EBCell2_P148 | CTCGGAACAACACTGCCACCGGGTTCTT | 28bp |
| EBCell2_P149 | CCGGTGGCAGTGTTGTTCCGAGCACGCA | 28bp |
| EBCell2_P150 | TTGCCTGATACCGGTGTATTTGCTGGCG | 28bp |
| EBCell2_P151 | AGCAAATACACCGGTATCAGGCAATTTGAAGGTTGA | 36bp |
| EBCell2_P152 | CCCCGACGCAATCAGCCACTCCAACGGTAACTCCTTCAGCCACCGCGACGCCTAC | 55bp |
| EBCell2_P155 | AATCGGATCCTAACGGGTTTGGCTTTGGCG | 30bp |
| EBCell2_P156 | CCAAACCCGTTAGGATCCGATTTACAGGTTGA | 32bp |
| EBCell2_P157 | TTATTTCCTGGTGAACTCGTTGGGGTATTAG | 31bp |
| EBCell2_P158 | CCCAACGAGTTCACCAGGAAATAAAATGAAAATTCAAATTGGTG | 44bp |
| EBCell2_P159 | GGTGAACTCGTTGGGGTATTAGTTGGCACTGAAGGGCGTGTCGGGGTCGCGGTTGCCGA | 59bp |
| EBCell2_P161 | ATTGCTTAAAGCGGAAATTGAACCAATTGCTTT | 33bp |
| EBCell2_P162 | GGTTCAATTTCCGCTTTAAGCAATAGTAAGTTAATACCTATT | 42bp |
| EBCell2_P164 | AGGATCCGATTTACAGGTTGA | 21bp |
| EBCell2_P165 | CCATGGGGTCAGACGGTGTG | 20bp |
| EBCell2_P166 | CTCGAGACTTGCAATTACCT | 20bp |
| EBCell2_P168 | GATCCCTCCTAGCGGATCCGATTTACAGGTTGACA | 35bp |
| EBCell2_P169 | AAATCGGATCCGCTAGGAGGGATCGTTGG | 29bp |
| EBCell2_P170 | TCCCTCCTAGCGGATCCGATTTACAGGTTGACA | 33bp |
| EBCell2_P171 | GCTAGGAGGGATCGTTGG | 18bp |
| EBCell2_P172 | GGTTGGTGTTGCCCCTTTCGTCGGTGTTGCATTGCCA | 37bp |
| EBCell2_P173 | CCGACGAAAGGGGCAACACCAACCA | 25bp |
| EBCell2_P174 | TTGCCTGATACCGGTGTATTTGCCGTTGG | 29bp |
| EBCell2_P175 | GGCAAATACACCGGTATCAGGCAATTTGAAGGTTGA | 36bp |
| EBCell2_P176 | TGTCAACCTGTAAATCGGATCCTAACGGGTTT | 32bp |
| EBCell2_P177 | AGGATCCGATTTACAGGTTGACATTGGAAGTACTA | 35bp |
| EBCell2_P178 | AAATCGGATCCTAACGGGTTTGGCTTTGGCGTACTGTTATTCGTAGGAGTCGA | 53bp |
| EBCell2_P179 | TTGGTGTTGCCCCTTTCGTCGGTGTTGCATTGCCA | 35bp |
| EBCell2_P180 | ACCGACGAAAGGGGCAACACCAA | 23bp |
| EBCell2_P181 | TCGGTGTTGCATTGCCAACGTTAA | 24bp |
| EBCell2_P182 | CGAAAGGGGCAACACCAA | 18bp |
| EBCell2_O183 | CGAAAGGGGCAACACCAACCAACACAGCCACGCCAACAAAGTCGGCAACCGCGACCCCGA | 60bp |
| EBCell2_P185 | CTCGGAACAACACTGCCACCGGGTTCTTTA | 30bp |
| EBCell2_P186 | ATTACTGTAACTAATACGCCGACCAATACTATCAGTG | 37bp |
| EBCell2_P187 | CCGACGAAAGGGGCAACACCAACCAAC | 27bp |
| EBCell2_P188 | TTGGTGTTGCCCCTTTCGTCGGTGTTGCATTGCCAACGTTAA | 42bp |
| EBCell2_P189 | CGAATAACAGTACGCCAAAGCCAAACCCGT | 30bp |
| EBCell2_P191 | GCCAAAGCCAAACCCGT | 17bp |
| EBCell2_P192 | GACAGGCTGCGTGCTCGGAACAACACTGC | 29bp |
| EBCell2_P193 | TGCTCGGAACAACACTGC | 18bp |
| EBCell2_P194 | CCGACGAAAGGGGCAACACCAA | 22bp |
| EBCell2_P195 | TCGGTGTTGCATTGCCAACGTT | 22bp |
| EBCell2_P196 | GTGTTGCATTGCCAACGTT | 19bp |
| EBCell2_P197 | GCCAAAGCCAAACCCGTTATCAGACG | 26bp |
| EBCell2_P198 | ATGGAACTCCTCTGAAATATACAGGTATTTCAACTGTAG | 39bp |
| EBCell2_P199 | CCTGTATATTTCAGAGGAGTTCCATCCAAAGGAATAG | 37bp |
| EBCell2_P200 | GCCAAACCCGTTATCAGACGGTGTGGTAGTAGA | 33bp |
| EBCell2_P203 | CGGAACAACACTGCCACC | 18bp |
| EBCell2_P204 | CAACCCCAGTAACCACACCA | 20bp |
| EBCell2_P205 | AAAAGCAGGTAGTGTTGTTAGTGTACCTATAACATTTACTAATGTACC | 48bp |
| EBCell2_O206 | CAACCCCAGTAACCACACCAACGATCCCTCCTAGCGGATCCGATTTACAGGTTGACATT | 59bp |
| EBCell2_P207 | TTGGTGTTGCCCCTTTCGTCGGTGTTGCATTGCCAACGTT | 40bp |
| EBCell2_P209 | TGTTCCGAGCACGCAGCCTGTCACTACGCCTCCGGCGACAACCCCAGTAACCACACCA | 58bp |
| EBCell2_P210 | TTGGCTTTGGCGTACTGTTATTCGTAGGAGTCGAATTGTTTGTCGGAG | 48bp |
| EBCell2_P211 | CGAATAACAGTACGCCAAAGCCAAACCCGTTAG | 33bp |
| EBCell2_P212 | AACACCGACGAAAGGGGCAACACCAA | 26bp |
| EBCell2_P213 | GGCTGCGTGCTCGGAACAACACTGCCACC | 29bp |
| EBCell2_P214 | ATCGGATCCCGTTGGGGTATTAGTTGGCACTG | 32bp |
| EBCell2_P215 | AGTGTTGTTCCGAGCACGCAGCCTGTCACTACGCCTCC | 38bp |
| EBCell2_P216 | TAATACCCCAACGGGATCCGATTTACAGGTTGACA | 35bp |
| EBCell2_P217 | CCATGGCAAATACACCGGTATCAGG | 25bp |
| EBCell2_P218 | TGCGTCGGGGTAGGACTTGCAATTACCTCAATTTTTC | 37bp |
| EBCell2_P220 | TTGGGGTATTAGTTGGCACTGAAGGGCG | 28bp |
| EBCell2_P221 | ACGGTGTGGTAGTAGAAATTGGCAAAGTTACGGG | 34bp |
| EBCell2_P222 | TAGAAATTGGCAAAGTTACGGG | 22bp |
| EBCell2_P224 | TGGTGTTGCCCCTTTCGTCGGTGTTGCATTGCCA | 34bp |
| EBCell2_P225 | TTCAGTGCCAACTAATACCCCAACGAGTTCACCAGGAAATAAAATGAAAAT | 51bp |
| EBCell2_P226 | CCATGGCAAATACACCGGTATCAGGCAA | 28bp |
| EBCell2_P227 | ACACCGTCTGATAACGGGTTTGGCTTTGGCG | 31bp |
| EBCell2_P229 | CAAACCCGTTATCAGACGGTGTGGTAGTAGAA | 32bp |
| EBCell2_P230 | CCTGGTGAACTGCTAGGAGGGATCGTTGG | 29bp |
| EBCell2_P231 | TCCCTCCTAGCAGTTCACCAGGAAATAAAATGAAAATTCAA | 41bp |
| EBCell2_P232 | CGGGTTCTTTACCCCATACAAGAACACCGTTCA | 33bp |
| EBCell2_P233 | TCTTGTATGGGGTAAAGAACCCGGTGGCAGT | 31bp |
| EBCell2_P234 | AAGAACCCGGTGGCAGTGTTGTTCCGAGCACGCAGCCTGTCACTACGCCTCCGG | 54bp |
| EBCell2_P235 | GCTAGGAGGGATCGTTGGTGTGGTTACTGGGGTTGTCGCCGGAGGCGTAGTGA | 53bp |
| EBCell2_P236 | AGTTCACCAGGAAATAAAATGAAAATTCAA | 30bp |
| EBCell2_P237 | AGTTCACCAGGAAATAAAATGAAAATTC | 28bp |
| EBCell2_P238 | TCCCTCCTAGCAGTTCACCAGGAAATAAAATGAAAATTC | 39bp |
| EBCell_P1 | CCATGGGGATCCGATTTACAG | 21bp |
| EBCell_P2 | CTCGAGACTGCCACCGGG | 18bp |
| EBCell_P3 | CCGGTGTATTTGCATTAGTTACAGTAATGCTTCCATCT | 38bp |
| EBCell_P4 | CATTACTGTAACTAATGCAAATACACCGGTATCAGGC | 37bp |
| EBCell_P5 | ATTAGTTACAGTAATGCTTCCATCT | 25bp |
| EBCell_P6 | CTGGTGAACTTGTTGCATTGCCAACGTTAA | 30bp |
| EBCell_P7 | GTTGGCAATGCAACAAGTTCACCAGGAAATAAAATGAAAATTC | 43bp |
| EBCell_P8 | TGTTGCATTGCCAACGTTAA | 20bp |
| EBCell_P9 | ACACCGTCGGAACTTGCAATTACCTCAATTTTTCCA | 36bp |
| EBCell_P10 | TAATTGCAAGTTCCGACGGTGTGGTAGTAG | 30bp |
| EBCell_P11 | CCATGGGGATCCGATTTACAGGTTGACA | 28bp |
| EBCell_P12 | ACTTGCAATTACCTCAATTTTTCCA | 25bp |
| EBCell_P13 | CTCGAGACTGCCACCGGGTTCTT | 23bp |
| EBCell_P14 | CCATGGTCCGACGGTGT | 17bp |
| EBCell_P15 | CTCGAGACTTGCAATTACCT | 20bp |
| EBCell_P16 | GGTGTATTTGCATTAGTTACAGTAATGCTTCCATC | 35bp |
| EBCell_P17 | GGAAGCATTACTGTAACTAATGCAAATACACCGGTATCAGGCAATTTGA | 49bp |
| EBCell_P18 | CATTAGTTACAGTAATGCTTCCATC | 25bp |
| EBCell_P19 | CCATGGTCCGACGGTGTGGTAGTAG | 25bp |
| EBCell_P20 | AGTTCACCAGGAAATAAAATGAAAATTC | 28bp |
| EBCell_P21 | CATTAGTTACAGTAATGCTTCCATCT | 26bp |
| EBCell_P22 | CAAATACACCGGTATCAGGCAATTTGA | 27bp |
| EBCell_P23 | AAATCGGATCCACTGCCACCGGGTTCTT | 28bp |
| EBCell_P24 | CCGGTGGCAGTGGATCCGATTTACAGGTTGACA | 33bp |
| EBCell_P25 | GGATCCGATTTACAGGTTGACA | 22bp |
| EBCell_P26 | CTCGAGACTTGCAATTACCTCAATTTTTCCA | 31bp |
| EBCell_P27 | CCATGGGCAAATACACCGGT | 20bp |
| EBCell_P28 | TCCTGGTGAACTTGTTGCATTGCCAACGTTAA | 32bp |
| EBCell_P29 | CAATGCAACAAGTTCACCAGGAAATAAAATGAAAATTC | 38bp |
| EBCell_P30 | CACACCGTCGGAACTGCCACCGGGTTCTT | 29bp |
| EBCell_P31 | CGGTGGCAGTTCCGACGGTGTGGTAGTAG | 29bp |
| EBCell_P32 | CCATGGGCAAATACACCGGTATCAGGC | 27bp |
| EBCell_P33 | ACTGCCACCGGGTTCTT | 17bp |
| EBCell_P34 | AAATCGGATCCATTAGTTACAGTAATGCTTCCATCT | 36bp |
| EBCell_P35 | GCATTACTGTAACTAATGGATCCGATTTACAGGTTGACA | 39bp |
| EBCell_P36 | CTCGAGATTAGTTACAGTAATGC | 23bp |
| EBCell_P37 | TCCTGGTGAACTACTGCCACCGGGTTCTT | 29bp |
| EBCell_P38 | CCGGTGGCAGTAGTTCACCAGGAAATAAAATGAAAATTC | 39bp |
| EBCell_P39 | CCGGTGTATTTGCTGTTGCATTGCCAACGTTAA | 33bp |
| EBCell_P40 | AATGCAACAGCAAATACACCGGTATCAGGC | 30bp |
| EBCell_P41 | CTCGAGATTAGTTACAGTAATGCTTCCATCT | 31bp |
| EBCell_P42 | CCACACCGTCGGAATTAGTTACAGTAATGCTTCCATCT | 38bp |
| EBCell_P43 | CATTACTGTAACTAATTCCGACGGTGTGGTAGTAG | 35bp |
| EBCell_P44 | TAATTGCAAGTAGTTCACCAGGAAATAAAATGAAAATTCAAATTGGTG | 48bp |
| EBCell_P45 | TCCGACGGTGTGGTAGTAG | 19bp |
| EBCell_P46 | GGTGTATTTGCTGTTGCATTGCCAACGTTAA | 31bp |
| EBCell_P47 | GCAATGCAACAGCAAATACACCGGTATCAGGC | 32bp |
| EBCell_P48 | TTATTTCCTGGTGAACTACTTGCAATTACCTCAATTTTTCCA | 42bp |
| EBCell_P49 | CTCGAGTGTTGCATTGCC | 18bp |
| EBCell_P50 | CGGTGTATTTGCATTAGTTACAGTAATGCTTCCATC | 36bp |
| EBCell_P51 | CATTACTGTAACTAATGCAAATACACCGGTATCAGGCAATTTGA | 44bp |
| EBCell_P52 | CTCGAGTGTTGCATTGCCAACGTTAA | 26bp |
| EBCell_P53 | ATTACCTCAATTTTTCCATCATTGTAAACAACATTTTTG | 39bp |
| EBCell_P54 | TTGTTTACAATGATGGAAAAATTGAGGTAATTGCAAGTGCA | 41bp |
| EBCell_P55 | ATTGAGGTAATTGCAAGTGCA | 21bp |
| EBCell_P56 | CAGATACTACTAACTCAATCAATCCTCAGTTCAAGGTTACT | 41bp |
| EBCell_P58 | GAGGATTGATTGAGTTAGTAGTATCTGAAGGATTGCTGTTGTAG | 44bp |
| EBCell_P59 | CCTGGTGAACTACTGCCACCGGGTTCTT | 28bp |
| EBCell_P60 | ACACCGTCGGAATTAGTTACAGTAATGCTTCCATCT | 36bp |
| EBCell_P61 | TACTGTAACTAATTCCGACGGTGTGGTAGTAG | 32bp |
| EBCell_P62 | AAATCGGATCCTGTTGCATTGCCAACGTTAA | 31bp |
| EBCell_P63 | GTTGGCAATGCAACAGGATCCGATTTACAGGTTGACA | 37bp |
| EBCell_P64 | CCATGGAGTTCACCAGGAAA | 20bp |
| EBCell_P65 | CCACACCGTCGGAACTTGCAATTACCTCAATTTTTCCA | 38bp |
| EBCell_P66 | CCATGGAGTTCACCAGGAAATAAAATGAAAATTC | 34bp |
| EBCell_P67 | ATTACTGTAACTAATTCCGACGGTGTGGTAGTAG | 34bp |
| EBCell_P68 | ATTACTGTAACTAATGCAAATACACCGGTATCAGGCAATTTGA | 43bp |
| EBCell_P69 | CGGTGTATTTGCATTAGTTACAGTAATGCTTCCATCT | 37bp |
| EBCell_P70 | GCAATGCAACAGGATCCGATTTACAGGTTGACA | 33bp |
| EBCell_P71 | CACACCGTCGGAACTTGCAATTACCTCAATTTTTCCA | 37bp |
| EBCell_P72 | AAATCGGATCCTGTTGCATTGCCAACGTTAAC | 32bp |
| EBCell_P73 | GGCAATGCAACAGGATCCGATTTACAGGTTGACA | 34bp |
| EBCell_P74 | TAGTTGGTGTATTAGTTACAGTAATGCTTCCATCT | 35bp |
| EBCell_P75 | AGCATTACTGTAACTAATACACCAACTAATACATCCGACG | 40bp |
| EBCell_P76 | ACACCAACACAGAGTTCACCAGGAAATAAAATGAAAATTC | 40bp |
| EBCell_P77 | CTGTGTTGGTGTTGGACTTGCAATTACCTCAATTTTTCCA | 40bp |
| EBCell_P78 | CCAAGGGAGCAGCAAATACACCGGTATCAGGC | 32bp |
| EBCell_P79 | TGCTCCCTTGGTCGGTGTTGCATTGCCAACGTTAA | 35bp |
| EBCell_P80 | ACACCAACTAATACATCCGACGGTGTGGTAGTAG | 34bp |
| EBCell_P81 | CCTGGTGAACTCTGTGTTGGTGTTGGACTTGCAATTACCTCAATTTTTCCA | 51bp |
| EBCell_P82 | GGTGTATTTGCTGCTCCCTTGGTCGGTGTTGCATTGCCAACGTTAA | 46bp |
| EBCell_P83 | AGCATTACTGTAACTAATACACCAACTAATACAGCAAATACAC | 43bp |
| EBCell_P84 | GATGGTACTACACTGCCACCGGGTTCTT | 28bp |
| EBCell_P85 | ACACCAACTAATACAGCAAATACACCGGT | 29bp |
| EBCell_P86 | GTAACCTTGAACTGAGGATTGATTGAGTTAGTAG | 34bp |
| EBCell_P87 | ACTCAATCAATCCTCAGTTCAAGGTTACTAATACCGGAAGC | 41bp |
| EBCell_P88 | CTAATACAGCAAATACACCGGTATCAGGC | 29bp |
| EBCell_P89 | GTAGTACCATCAACATCCGACGGTGTGGTAGTAG | 34bp |
| EBCell_P90 | CCGGTGGCAGTGTAGTACCATCAACATCCGACGGTGTGGTAGTAG | 45bp |
| EBCell_P91 | ATGGTACTACACTGCCACCGGGTTCTT | 27bp |
| EBCell_P92 | CCCGGTGGCAGTGTAGTACCATCAACATCCGACG | 34bp |
| EBCell_P93 | ACCAACACAGGCAAATACACCGGTATCAGGC | 31bp |
| EBCell_P94 | GACCAAGGGAGCAAGTTCACCAGGAAATAAAATGAAAATTC | 41bp |
| EBCell_P95 | CGGTGTATTTGCCTGTGTTGGTGTTGGACTTGCAATTACCTCAATTTTTCCA | 52bp |
| EBCell_P96 | CTGGTGAACTTGCTCCCTTGGTCGGTGTTGCATTGCCAACGTTAA | 45bp |
| EBCell_P97 | CCCGGTGGCAGTGTAGTACCATCAACAAGTTCACCA | 36bp |
| EBCell_P98 | GTAGTACCATCAACAAGTTCACCAGGAAATAAAATGAAAATTC | 43bp |
| EBCell_P99 | AGCATTACTGTAACTAATACACCAACTAATACATCCGACGGTGTGGTAGTAG | 52bp |
| EBCell_P100 | AGTTGGTGTATTAGTTACAGTAATGCTTCCATCT | 34bp |
| EBCell_P101 | CCCTTGGTCGGTGTTGCATTGCCAACGTTAA | 31bp |
| EBCell_P102 | CCGACCAAGGGAGCAGGATCCGATTTACAGGTTGACA | 37bp |
| EBCell_P103 | CCAACACCAACACAGAGTTCACCAGGAAATAAAATGAAAATTC | 43bp |
| EBCell_P104 | GCAATGCAACACCGACCAAGGGAGCAGGATCCGATTTACAGGTTGACA | 48bp |
| EBCell_P105 | TGCAAGTCCAACACCAACACAGAGTTCACCAGGAAATAAAATGAAAATTC | 50bp |
| EBCell_P106 | AGCATTACTGTAACTAATACACCAACTAATACAGCAAATACACCGGT | 47bp |
| EBCell_P107 | CTTGTTGATGGTACTACACTGCCACCGGGTTCTT | 34bp |
| EBCell_P108 | CCTGTGTTGGTGTTGGACTTGCAATTACCTCAATTTTTCCA | 41bp |
| EBCell_P109 | CCAACACCAACACAGGCAAATACACCGGT | 29bp |
| EBCell_P110 | TGCAAGTCCAACACCAACACAGGCAAATACACCGGT | 36bp |
| EBCell_P111 | TGGCAGTGTAGTACCATCAACAAGTTCACCAGGAAATAAAATGAAAATTC | 50bp |
| EBCell_P112 | AAATCGGATCCTGTATTAGTTGGTGTATTAGTTACAGTAA | 40bp |
| EBCell_P113 | CACCAACTAATACAGGATCCGATTTACAGGTTGACA | 36bp |
| EBCell_P114 | CCGGTGTATTTGCTGTATTAGTTGGTGTATTAGTTACAG | 39bp |
| EBCell_P115 | CAACTAATACAGCAAATACACCGGTATCAGGCAATTTGA | 39bp |
| EBCell_P116 | CTGTATTAGTTGGTGTATTAGTTACAGTAATGCTTCCATCT | 41bp |
| EBCell_P117 | GTAGTACCATCAACAGGATCCGATTTACAGGTTGACA | 37bp |
| EBCell_P118 | CCGGTGGCAGTGTAGTACCATCAACAGGATCCGATTTACAGGTTGACA | 48bp |
| EBCell_P119 | CTACACTGCCACCGGGTTCTT | 21bp |
| EBCell_P120 | GGTAAAGAACCCGGTGGCAGTGTAGTACCATCAACAGGATCCG | 43bp |
| EBCell_P121 | TGTGTTGGTGTTGGACTTGCAATTACCTCAATTTTTCCA | 39bp |
| EBCell_P122 | TAATTGCAAGTCCAACACCAACACAGAGTTCA | 32bp |
| EBCell_P123 | TAATTGCAAGTCCAACACCAACACAGAGTTCACCAGGAAATAAAATGAAAATTC | 54bp |
| EBCell_P124 | CTTGTTGATGGTACTACACTGCCACCGGGTTC | 32bp |
| EBCell_P125 | TGGCAGTGTAGTACCATCAACAAGTTCACCAG | 32bp |
| EBCell_P126 | ACACTGCCACCGGGTTCTT | 19bp |
| EBCell_P127 | AGTACCATCAACAAGTTCACCAGGAAATAAAATGAAAATTC | 41bp |
| EBCell_P128 | GGAAGCATTACTGTAACTAATACACCAACTAATACAGGATCCGATTTACAGGTTGACA | 58bp |
| EBCell_P129 | TAATTGCAAGTCCAACACCAACACAGGCAAATAC | 34bp |
| EBCell_P130 | AAATCGGATCCTGCTCCCTTGGTCGGTG | 28bp |
| EBCell_P131 | CCAAGGGAGCAGGATCCGATTTACAGGTTGACA | 33bp |
| EBCell_P132 | CCAACTAATACATCCGACGGTGTGGTAGTAG | 31bp |
| EBCell_P133 | ACACCGTCGGATGTATTAGTTGGTGTATTAGTTACAGTAATGCTTCCATCT | 51bp |
| EBCell_P134 | CCACACCGTCGGATGTATTAGTTGGTGTATTAGTTACAGTAATGCTTCCATCT | 53bp |
| EBCell_P135 | CCAACTAATACAGCAAATACACCGGTATCAGGCAATTTGA | 40bp |
| EBCell_P136 | GTGTAGTACCATCAACATCCGACGGTGTGGTAGTAG | 36bp |
| EBCell_P137 | AAATCGGATCCTGCTCCCTTGGTCGGTGTTGCATTGCCAACGTTAA | 46bp |
| EBCell_P138 | CGTCGGATGTTGATGGTACTACACTGCCACCGGGTTCTT | 39bp |
| EBCell_P139 | CCGGTGTATTTGCTGTATTAGTTGGTGTATTAGTTACAGTAATGCTTCCATCT | 53bp |
| EBCell_P140 | GTTGGCAATGCAACACCGACCAAGGGAGCAG | 31bp |
| EBCell_P141 | GTTGGCAATGCAACACCGACCAAGGGAGCAAG | 32bp |
| EBCell_P142 | CCGACCAAGGGAGCAAGTTCACCAGGAAATAAAATGAAAATTC | 43bp |
| EBCell_P143 | GTGTAGTACCATCAACAAGTTCACCAGGAAATAAAATGAAAATTC | 45bp |
| EBCell_P144 | CCCTTGGTCGGTGTTGCATTGCCAACGTTAAC | 32bp |
| EBCell_P145 | GGCAATGCAACACCGACCAAGGGAGCAGGATCCGATTTACAGGTTGACA | 49bp |
| EBCell_P146 | CCTGGTGAACTTGTTGATGGTACTACACTGCCACCGGGTTCTT | 43bp |
