# Supplementary material for: A synthetic biology approach for evaluating the functional contribution of designer cellulosome components to deconstruction of cellulosic substrates
Source: Biotechnol Biofuels. 2013 Dec 16;6:182. doi: 10.1186/1754-6834-6-182 (PMC3878649; doi:10.1186/1754-6834-6-182)
Supplement: Additional file 6: Table S4 — Primer sequences used for the cloning of the long inter-modular linker scaffoldins. Modules are color-coded: plasmid sequences (red), Coh T (black), carbohydrate binding module (CBM) (c)(brown), Coh B (green), Coh A (magenta). Primer sequences that appear in boldface are primers that already appear in the list in the primer set of a former scaffoldin. [file 1754-6834-6-182-S6.docx]

| **Construct** | **Modular composition** | **Primer name** | **Primer seuquence** |
| --- | --- | --- | --- |
| **Scaf9L:** | T-lT-B-lB-A-lA-CBM |  |  |
|  |  | NcoI_pET28_T_F | ctttaagaaggagatataccatgggctcCgacggtgtggtagtagaaa |
|  |  | T-linker_B_R comp&rev | GAATTTTCATTTTATTTCCTGGTGAACTCGGTGTGTTTGTCGGTGTGTTTG |
|  |  | T-linker_B_F | GAcaaacacaccgacaaacacaccgagttcaccaggaaataaaatgaaaattc |
|  |  | B-linker_A_R comp&rev | CCAATGTCAACCTGTAAATCGGATCCTAACGGGTTTGGCTTTGGCGTAC |
|  |  | B-linker_A_F | cagtacgccaaagccaaacccgttaggatccgatttacaggttgacattg |
|  |  | A-linker_CBM_R comp&rev | CAAATTGCCTGATACCGGTGTATTTGCTGGCGTTACAGTCGGCGTAGC |
|  |  | A-linker_CBM_F | gagtgctacgccgactgtaacgccagcaaatacaccggtatcaggc |
|  |  | CBM_XhoI_R comp&rev | CAGTGGTGGTGGTGGTGGTGCTCGAGTTAACTGCCACCGGGTTCTTTACC |
| **Scaf12L:** | T-lT-CBM-lCBM-B-lB-A |  |  |
|  |  | **NcoI_pET28_T_F** | ctttaagaaggagatataccatgggctcCgacggtgtggtagtagaaa |
|  |  | CBM-linker_B_R comp&rev | GAATTTTCATTTTATTTCCTGGTGAACTTGACGGCGGTATTGTTGTTGCAGG |
|  |  | CBM-linker_B_F | ccacctgcaacaacaataccgccgtcaagttcaccaggaaataaaatgaaaattc |
|  |  | A_XhoI_R comp&rev | CAGTGGTGGTGGTGGTGGTGCTCGAGTAAACTTGCAATTACCTCAATTTTTCC |
| **Scaf10L:** | T-lT-B-lB-CBM- lCBM-A |  |  |
|  |  | **NcoI_pET28_T_F** | ctttaagaaggagatataccatgggctcCgacggtgtggtagtagaaa |
|  |  | **T-linker_B_R comp&rev** | GAATTTTCATTTTATTTCCTGGTGAACTCGGTGTGTTTGTCGGTGTGTTTG |
|  |  | **T-linker_B_F** | GAcaaacacaccgacaaacacaccgagttcaccaggaaataaaatgaaaattc |
|  |  | B-linker_CBM_R comp&rev | CAAATTGCCTGATACCGGTGTATTTGCTAACGGGTTTGGCTTTGGCGTAC |
|  |  | B-linker -CBM_F | cagtacgccaaagccaaacccgttagcaaatacaccggtatcaggc |
|  |  | CBM-linker_A_R comp&rev | CCAATGTCAACCTGTAAATCGGATCCTGACGGCGGTATTGTTGTTGC |
|  |  | CBM-linker_A_F | aacaacaataccgccgtcaggatccgatttacaggttgacat |
|  |  | **A_XhoI_R comp&rev** | CAGTGGTGGTGGTGGTGGTGCTCGAGACTTGCAATTACCTCAATTTTTCC |
| **Scaf22L:** | CBM-lCBM-C-lC-B-lB-A |  |  |
|  |  | NcoIpET28_CBM_F | ctttaagaaggagatataccatggcaaatacaccggtatcaggcaat |
|  |  | CBM-linker_T_R comp&rev | CCAATTTCTACTACCACACCGTCGGATGACGGCGGTATTGTTGTTGCAG |
|  |  | CBM-linker_T_F | CACctgcaacaacaataccgccgtcatcCgacggtgtggtagtagaaa |
|  |  | **T-linker_B_R comp&rev** | GAATTTTCATTTTATTTCCTGGTGAACTCGGTGTGTTTGTCGGTGTGTTTG |
|  |  | **T-linker_B_F** | GAcaaacacaccgacaaacacaccgagttcaccaggaaataaaatgaaaattc |
|  |  | **B-linker_A_R comp&rev** | CCAATGTCAACCTGTAAATCGGATCCTAACGGGTTTGGCTTTGGCGTAC |
|  |  | **B-linker_A_F** | cagtacgccaaagccaaacccgttaggatccgatttacaggttgacattg |
|  |  | A_XhoI_R_comp&rev | CAGTGGTGGTGGTGGTGGTGCTCGAGACTTGCAATTACCTCAATTTTTCC |
| **Scaf5L:** | A-lA-CBM-lCBM-T-lT-B |  |  |
|  |  | NcoIpET28_A_F | ctttaagaaggagatataccatgggatccgatttacaggttgacat |
|  |  | **A-linker_CBM_R comp&rev** | CAAATTGCCTGATACCGGTGTATTTGCTGGCGTTACAGTCGGCGTAGC |
|  |  | **A-linker_CBM_F** | gagtgctacgccgactgtaacgccagcaaatacaccggtatcaggc |
|  |  | **CBM-linker_T_R comp&rev** | CCAATTTCTACTACCACACCGTCGGATGACGGCGGTATTGTTGTTGCAG |
|  |  | **CBM-linker_T_F** | CACctgcaacaacaataccgccgtcatcCgacggtgtggtagtagaaa |
|  |  | **T-linker_B_R comp&rev** | GAATTTTCATTTTATTTCCTGGTGAACTCGGTGTGTTTGTCGGTGTGTTTG |
|  |  | **T-linker_B_F** | GAcaaacacaccgacaaacacaccgagttcaccaggaaataaaatgaaaattc |
|  |  | B_XhoI_R comp&rev | CAGTGGTGGTGGTGGTGGTGCTCGAGATTAGTTACAGTAATGCTTCCATC |
| **Scaf6L:** | A-lA-CBM-lCBM-B-lB-T |  |  |
|  |  | **NcoIpET28**_A_F | ctttaagaaggagatataccatgggatccgatttacaggttgacat |
|  |  | **A-linker_CBM_R comp&rev** | CAAATTGCCTGATACCGGTGTATTTGCTGGCGTTACAGTCGGCGTAGC |
|  |  | **A-linker_CBM_F** | gagtgctacgccgactgtaacgccagcaaatacaccggtatcaggc |
|  |  | **CBM-linker_B_R comp&rev** | GAATTTTCATTTTATTTCCTGGTGAACTTGACGGCGGTATTGTTGTTGCAGG |
|  |  | CBMl-inker_B_F | ccacctgcaacaacaataccgccgtcaagttcaccaggaaataaaatgaaaattc |
|  |  | B-linker_T_R comp&rev | CCAATTTCTACTACCACACCGTCGGATAACGGGTTTGGCTTTGGCGTAC |
|  |  | B-linker_T_F | cagtacgccaaagccaaacccgttatcCgacggtgtggtagtagaaa |
|  |  | T_Xho_R comp&rev | CAGTGGTGGTGGTGGTGGTGCTCGAGTGTTGCATTGCCAACGTTAACAC |
| **Scaf11L:** | T-lT-CBM-lCBM-A-lA-B |  |  |
|  |  | **NcoI_pET28_T_F** | ctttaagaaggagatataccatgggctcCgacggtgtggtagtagaaa |
|  |  | **CBM-linker_A_R comp&rev** | CCAATGTCAACCTGTAAATCGGATCCTGACGGCGGTATTGTTGTTGC |
|  |  | **CBM-linker_A_F** | aacaacaataccgccgtcaggatccgatttacaggttgacat |
|  |  | A-linker_B_R comp&rev | GAATTTTCATTTTATTTCCTGGTGAACTTGGCGTTACAGTCGGCGTAGC |
|  |  | A-linker_B_F | gagtgctacgccgactgtaacgccaagttcaccaggaaataaaatgaaaattc |
|  |  | B_XhoI_R comp&rev | CAGTGGTGGTGGTGGTGGTGCTCGAGATTAGTTACAGTAATGCTTCCATC |
| **Scaf17L:** | B-lB-CBM- lCBM-A-lA-T |  |  |
|  |  | NcoIpET28_B_F | ctttaagaaggagatataccatgggcagttcaccaggaaataaaatgaaaattc |
|  |  | B-linker_CBM_R comp&rev | CAAATTGCCTGATACCGGTGTATTTGCTAACGGGTTTGGCTTTGGCGTAC |
|  |  | B-linker -CBM_F | cagtacgccaaagccaaacccgttagcaaatacaccggtatcaggc |
|  |  | **CBM-linker_A_R comp&rev** | CCAATGTCAACCTGTAAATCGGATCCTGACGGCGGTATTGTTGTTGC |
|  |  | **CBM-linker_A_F** | aacaacaataccgccgtcaggatccgatttacaggttgacat |
|  |  | A-linker_T_R comp&rev | CCAATTTCTACTACCACACCGTCGGATGGCGTTACAGTCGGCGTAGC |
|  |  | A-linker_T_F | gagtgctacgccgactgtaacgccatcCgacggtgtggtagtagaaa |
|  |  | T_Xho_R comp&rev | CAGTGGTGGTGGTGGTGGTGCTCGAGTGTTGCATTGCCAACGTTAACAC |
| **Scaf18L:** | B-lB-CBM- lCBM-T-lT-A |  |  |
|  |  | NcoIpET28_B_F | ctttaagaaggagatataccatgggcagttcaccaggaaataaaatgaaaattc |
|  |  | B-linker_CBM_R comp&rev | CAAATTGCCTGATACCGGTGTATTTGCTAACGGGTTTGGCTTTGGCGTAC |
|  |  | B-linker -CBM_F | cagtacgccaaagccaaacccgttagcaaatacaccggtatcaggc |
|  |  | **CBM-linker_T_R comp&rev** | CCAATTTCTACTACCACACCGTCGGATGACGGCGGTATTGTTGTTGCAG |
|  |  | **CBM-linker_T_F** | CACctgcaacaacaataccgccgtcatcCgacggtgtggtagtagaaa |
|  |  | T-linker -A_R comp&rev | CCAATGTCAACCTGTAAATCGGATCCCGGTGTGTTTGTCGGTGTGTTT  G |
|  |  | T-linker -A_F | GAcaaacacaccgacaaacacaccgggatccgatttacaggttgacattg |
|  |  | **A_XhoI_R comp&rev** | CAGTGGTGGTGGTGGTGGTGCTCGAGACTTGCAATTACCTCAATTTTTCC |
| **Scaf3L** | A-lA- B-lB- T-lT-CBM | **NcoIpET28_A_F** | ctttaagaaggagatataccatgggatccgatttacaggttgacat |
|  |  | **A-linker_B_R comp&rev** | GAATTTTCATTTTATTTCCTGGTGAACTTGGCGTTACAGTCGGCGTAGC |
|  |  | **A-linker_B_F** | gagtgctacgccgactgtaacgccaagttcaccaggaaataaaatgaaaattc |
|  |  | **B-linker_T_R comp&rev** | CCAATTTCTACTACCACACCGTCGGATAACGGGTTTGGCTTTGGCGTAC |
|  |  | **B-linker_T_F** | cagtacgccaaagccaaacccgttatcCgacggtgtggtagtagaaa |
|  |  | **CBM_XhoI_R comp&rev** | CAGTGGTGGTGGTGGTGGTGCTCGAGTTAACTGCCACCGGGTTCTTTACC |
| **Scaf4L** | A-lA-B-lB-CBM-lCBM-T | **NcoIpET28_A_F** | ctttaagaaggagatataccatgggatccgatttacaggttgacat |
|  |  | **A-linker_B_R comp&rev** | GAATTTTCATTTTATTTCCTGGTGAACTTGGCGTTACAGTCGGCGTAGC |
|  |  | **A-linker_B_F** | gagtgctacgccgactgtaacgccaagttcaccaggaaataaaatgaaaattc |
|  |  | **B-linker_CBM_R comp&rev** | CAAATTGCCTGATACCGGTGTATTTGCTAACGGGTTTGGCTTTGGCGTAC |
|  |  | **B-linker -CBM_F** | cagtacgccaaagccaaacccgttagcaaatacaccggtatcaggc |
|  |  | **CBM-linker_T_R comp&rev** | CCAATTTCTACTACCACACCGTCGGATGACGGCGGTATTGTTGTTGCAG |
|  |  | **CBM-linker_T_F** | CACctgcaacaacaataccgccgtcatcCgacggtgtggtagtagaaa |
|  |  | **T_Xho_R comp&rev** | CAGTGGTGGTGGTGGTGGTGCTCGAGTGTTGCATTGCCAACGTTAACAC |
| Scaf19L | CBM-lCBM-A-lA-C-lC-B |  |  |
|  |  | **NcoIpET28_CBM_F** | ctttaagaaggagatataccatggcaaatacaccggtatcaggcaat |
|  |  | **CBM-linker_A_R comp&rev** | CCAATGTCAACCTGTAAATCGGATCCTGACGGCGGTATTGTTGTTGC |
|  |  | **CBM-linker_A_F** | aacaacaataccgccgtcaggatccgatttacaggttgacat |
|  |  | **A-linker_T_R comp&rev** | CCAATTTCTACTACCACACCGTCGGATGGCGTTACAGTCGGCGTAGC |
|  |  | **A-linker_T_F** | gagtgctacgccgactgtaacgccatcCgacggtgtggtagtagaaa |
|  |  | **T-linker_B_R comp&rev** | GAATTTTCATTTTATTTCCTGGTGAACTCGGTGTGTTTGTCGGTGTGTTTG |
|  |  | **T-linker_B_F** | GAcaaacacaccgacaaacacaccgagttcaccaggaaataaaatgaaaattc |
|  |  | **B_XhoI_R comp&rev** | CAGTGGTGGTGGTGGTGGTGCTCGAGATTAGTTACAGTAATGCTTCCATC |
| **Scaf20L** | CBM-lCBM-A-lA-B-lB- T |  |  |
|  |  | **NcoIpET28_CBM_F** | ctttaagaaggagatataccatggcaaatacaccggtatcaggcaat |
|  |  | **CBM-linker_A_R comp&rev** | CCAATGTCAACCTGTAAATCGGATCCTGACGGCGGTATTGTTGTTGC |
|  |  | **CBM-linker_A_F** | aacaacaataccgccgtcaggatccgatttacaggttgacat |
|  |  | **A-linker_B_R comp&rev** | GAATTTTCATTTTATTTCCTGGTGAACTTGGCGTTACAGTCGGCGTAGC |
|  |  | **A-linker_B_F** | gagtgctacgccgactgtaacgccaagttcaccaggaaataaaatgaaaattc |
|  |  | **B-linker_T_R comp&rev** | CCAATTTCTACTACCACACCGTCGGATAACGGGTTTGGCTTTGGCGTAC |
|  |  | **B-linker_T_F** | cagtacgccaaagccaaacccgttatcCgacggtgtggtagtagaaa |
|  |  | **T_Xho_R comp&rev** | CAGTGGTGGTGGTGGTGGTGCTCGAGTGTTGCATTGCCAACGTTAACAC |
| **Scaf21L** | CBM-lCBM**-**T-lT-A-lA-B | **NcoIpET28_CBM_F** | ctttaagaaggagatataccatggcaaatacaccggtatcaggcaat |
|  |  | **CBM-linker_T_R comp&rev** | CCAATTTCTACTACCACACCGTCGGATGACGGCGGTATTGTTGTTGCAG |
|  |  | **CBM-linker_T_F** | CACctgcaacaacaataccgccgtcatcCgacggtgtggtagtagaaa |
|  |  | **T-linker -A_R comp&rev** | CCAATGTCAACCTGTAAATCGGATCCCGGTGTGTTTGTCGGTGTGTTT  G |
|  |  | **T-linker -A_F** | GAcaaacacaccgacaaacacaccgggatccgatttacaggttgacattg |
|  |  | **A-linker_B_R comp&rev** | GAATTTTCATTTTATTTCCTGGTGAACTTGGCGTTACAGTCGGCGTAGC |
|  |  | **A-linker_B_F** | gagtgctacgccgactgtaacgccaagttcaccaggaaataaaatgaaaattc |
|  |  | **B_XhoI_R comp&rev** | CAGTGGTGGTGGTGGTGGTGCTCGAGATTAGTTACAGTAATGCTTCCATC |
| **Scaf23L** | CBM-lCBM**-**B-A-lA**-**T | **NcoIpET28_CBM_F** | ctttaagaaggagatataccatggcaaatacaccggtatcaggcaat |
|  |  | **CBM-linker_B_R comp&rev** | GAATTTTCATTTTATTTCCTGGTGAACTTGACGGCGGTATTGTTGTTGCAGG |
|  |  | **CBM-linker_B_F** | ccacctgcaacaacaataccgccgtcaagttcaccaggaaataaaatgaaaattc |
|  |  | **B-linker_A_R comp&rev** | CCAATGTCAACCTGTAAATCGGATCCTAACGGGTTTGGCTTTGGCGTAC |
|  |  | **B-linker_A_F** | cagtacgccaaagccaaacccgttaggatccgatttacaggttgacattg |
|  |  | **A-linker_T_R comp&rev** | CCAATTTCTACTACCACACCGTCGGATGGCGTTACAGTCGGCGTAGC |
|  |  | **A-linker_T_F** | gagtgctacgccgactgtaacgccatcCgacggtgtggtagtagaaa |
|  |  | **T_Xho_R comp&rev** | CAGTGGTGGTGGTGGTGGTGCTCGAGTGTTGCATTGCCAACGTTAACAC |
| **Scaf24L** | CBM-lCBM-B-lB-T-lT-A |  |  |
|  |  | **NcoIpET28_CBM_F** | ctttaagaaggagatataccatggcaaatacaccggtatcaggcaat |
|  |  | **CBM-linker_B_R comp&rev** | GAATTTTCATTTTATTTCCTGGTGAACTTGACGGCGGTATTGTTGTTGCAGG |
|  |  | **CBM-linker_B_F** | ccacctgcaacaacaataccgccgtcaagttcaccaggaaataaaatgaaaattc |
|  |  | **B-linker_T_R comp&rev** | CCAATTTCTACTACCACACCGTCGGATAACGGGTTTGGCTTTGGCGTAC |
|  |  | **B-linker_T_F** | cagtacgccaaagccaaacccgttatcCgacggtgtggtagtagaaa |
|  |  | **T-linker -A_R comp&rev** | CCAATGTCAACCTGTAAATCGGATCCCGGTGTGTTTGTCGGTGTGTTT  G |
|  |  | **T-linker -A_F** | GAcaaacacaccgacaaacacaccgggatccgatttacaggttgacattg |
|  |  | **A_XhoI_R comp&rev** | CAGTGGTGGTGGTGGTGGTGCTCGAGACTTGCAATTACCTCAATTTTTCC |
